# Capacitive interdigitated system of high osteoinductive/conductive performance for personalized acting-sensing implants

**DOI:** 10.1038/s41536-021-00184-6

**Published:** 2021-11-23

**Authors:** Bárbara M. de Sousa, Clara R. Correia, Jorge A. F. Ferreira, João F. Mano, Edward P. Furlani, Marco P. Soares dos Santos, Sandra I. Vieira

**Affiliations:** 1 Department of Medical Sciences, Institute of Biomedicine (iBiMED), University of Aveiro, 3810-193 Aveiro, Portugal; 2 Department of Chemistry, CICECO – Aveiro Institute of Materials, University of Aveiro, 3810-193 Aveiro, Portugal; 3 Department of Mechanical Engineering, Centre for Mechanical Technology & Automation (TEMA), University of Aveiro, 3810-193 Aveiro, Portugal; 4 Department of Chemical and Biological Engineering, Department of Electrical Engineering, University at Buffalo (SUNY), Buffalo, NY 14260 USA; 5 Faculty of Engineering, Associated Laboratory for Energy, Transports and Aeronautics (LAETA), University of Porto, 4200-465 Porto, Portugal

**Keywords:** Fracture repair, Regenerative medicine, Bone, Implants, Bone

## Abstract

Replacement orthopedic surgeries are among the most common surgeries worldwide, but clinically used passive implants cannot prevent failure rates and inherent revision arthroplasties. Optimized non-instrumented implants, resorting to preclinically tested bioactive coatings, improve initial osseointegration but lack long-term personalized actuation on the bone–implant interface. Novel bioelectronic devices comprising biophysical stimulators and sensing systems are thus emerging, aiming for long-term control of peri-implant bone growth through biointerface monitoring. These acting-sensing dual systems require high frequency (HF) operations able to stimulate osteoinduction/osteoconduction, including matrix maturation and mineralization. A sensing-compatible capacitive stimulator of thin interdigitated electrodes and delivering an electrical 60 kHz HF stimulation, 30 min/day, is here shown to promote osteoconduction in pre-osteoblasts and osteoinduction in human adipose-derived mesenchymal stem cells (hASCs). HF stimulation through this capacitive interdigitated system had significant effects on osteoblasts’ collagen-I synthesis, matrix, and mineral deposition. A proteomic analysis of microvesicles released from electrically-stimulated osteoblasts revealed regulation of osteodifferentiation and mineralization-related proteins (e.g. Tgfb3, Ttyh3, Itih1, Aldh1a1). Proteomics data are available via ProteomeXchange with the identifier PXD028551. Further, under HF stimulation, hASCs exhibited higher osteogenic commitment and enhanced hydroxyapatite deposition. These promising osteoinductive/conductive capacitive stimulators will integrate novel bioelectronic implants able to monitor the bone–implant interface and deliver personalized stimulation to peri-implant tissues.

## Introduction

Biophysical stimulation is recognized as a forthcoming therapeutic for various diseases, including musculoskeletal disorders^1^, and upcoming advanced multifunctional medical devices will certainly profit from biophysical stimulation for targeted and long-lasting therapies^2–7^. Both primary and revision hip/knee arthroplasties are examples of interventions of high socio-economic cost that can benefit from such therapies. The most common indicator for these joint replacements is osteoarthritis, currently affecting 7% of the global population and whose global prevalence increased by ~50% in three decades, alongside longevity and sedentarism^8^. Adding to the increased number of primary surgeries, up to 10% of patients still face implant failures from dislocation or aseptic loosening^9–13^. Currently, around 30% of patients experiencing such failures are young, a number expected to doble in 10 years^14–16^. Failure rates are two-fold higher for young patients^17^, who present a 35% revision risk in the 40-year follow-up upon cemented hip arthroplasties^18^. Implant technologies using uncemented fixations are thus significantly increasing worldwide^19^, particularly for younger and active patients whose lifestyle places heavy demands on implants^20^. Cementless fixations aim to maximize implant survival and outperform the mid- and long-term high deterioration risk of cemented implants^21,22^. Nevertheless, the risk of bone loss is higher due to stress-shielding using uncemented fixations, given the reduced mechanical stimuli delivered to peri-implant bone structures upon implant insertion^6,20,21^. Unstable bone–implant fixations triggered by stress-shielding can induce aseptic loosening, an adverse bone remodeling response that exceeds 50% of the revision indications^6,9,10,23^. Implantable systems that significantly reduce bone–implant integration failures are therefore highly demanded.

Strategies to improve the performance of non-instrumented implants have focused on optimizing geometry and materials^13,21,22,24^. However, the innovative advances on custom-made geometries, nanometer-scale textured surfaces, and porous or multi-material structures^21,22,25–29^ are passive approaches, not sufficient to avoid osseointegration failures^7^. (Bio)chemical surface modifications, allowing to design non-instrumented active implants, are more effective^22,30–33^. Innovative bioactive coatings with drug-releasing or bio-agent properties can improve initial bone-implant integration, as highlighted by preclinical results^2,19,22,30–32,34^. These technologies open attractive opportunities to enhance osseointegration, but are limited in long-term implant survival and controllability since: (i) they are not dynamically delivered considering the bone–implant interface state; (ii) their actuation cannot be modified after implant insertion; (iii) their ability to deliver personalized therapeutic stimuli to specific implant–bone interface regions is low or null^3,6^.

New bioelectronic implantable devices have been designed to overcome these issues^3,6,7,35,36^. So far, only instrumented passive bioelectronic devices have been implanted in humans to monitor the implant’s biomechanical properties (mainly forces, deformations, and temperatures), and communicate data to extracorporeal systems^13,24,37–40^. Aiming to deliver highly controlled and personalized therapeutic actuations to peri-implant tissues, a new era in bone implant technology is emerging^3,6,7,13^. Aligned with this is our vision of multifunctional bioelectronic implants incorporating acting-sensing dual systems, where electrodes are used for both biophysical actuation and monitoring bone-implant fixation. These implants will further include self-powering systems and wireless communication, so clinicians can control their operation extracorporeally^3,6,7,13,36,41^.

Electrical stimulation, studied for bone healing since the first bone piezoelectricity reports^42,43^, may be delivered through direct current (DC), inductive coupling (IC), or capacitive coupling (CC). This last requires two electrodes to generate and deliver electric fields (EFs)^44^. Contrary to DC and IC, CC stimulation induces less reactive oxygen species or oxidative stress^44^, while promoting osteogenic effects in vitro^45–49^, either using low frequencies (LFs)^45,46^ or high frequencies (HFs)^47–49^. CC stimulation with HF (60 kHz) can also aid in bone fracture healing in vivo^50–53^. However, currently in orthopedics, CC uses extracorporeal electrodes placed parallelly on opposite sides of the bone. This configuration requires very high-voltage excitations for bone stimulation and ends up impacting non-target tissues, as muscle and nerves^4^. Further, these parallel electrodes cannot be incorporated into intracorporeal implants, as generated EFs would be circumscribed inside the implant and, consequently, would not deliver stimuli to the bone–implant interface, having no therapeutic effect on the fracture^6^. Also, despite reported efficacy of parallel CC stimulators for bone healing, their extracorporeal actuation cannot ensure long-lasting effective bone–implant fixation.

Our groundbreaking solution is based on “cosurface” architectures, with positively-charged and grounded electrodes on the same surface, for incorporation on implant’s surface for targeted bone stimulation^6^. These cosurface capacitive electrodes can enhance collagen synthesis via contactless bioelectrical stimulation of osteoblasts^3,6,54^. However, our first-developed cosurface electrodes (striped pattern) had no impact on alkaline phosphatase (ALP) secretion or extracellular matrix mineralization, using either LF or HF stimuli^6^. Besides, their large thickness compromises incorporation into instrumented implants^6^. A following work, where various capacitive architectures were tested, revealed a strong dependency of osteogenic effects on electrode specificities, as reduced thickness^3,54^. Indeed, our 100-µm-thick interdigitated architecture delivering LF stimulation positively impacted collagen and ALP^3^. However, reduced thickness and LF stimulation improved mostly matrix maturation, but did not enhance mineralization^3^. Indeed, none of the electrical stimulation set-ups tested so far using cosurface electrodes had positive osteoconductive effects on both matrix maturation and mineralization phases (and their osteoinductive ability had been never tested). Moreover, LF stimulation is not compatible with sensing technologies to monitor loosening states, desirable for future bioelectronic implants^23^. Contrarily, our recent monitoring assays with bone blocks revealed that capacitive cosurface architectures powered by HF excitations can detect macro- and microscale interface loosening^36^.

In sum, we here propose a capacitive stimulation system combining: (i) the osteogenic effects promoted by microscale-thickness cosurface electrodes; (ii) the structural advantage of easily incorporating such electrodes below the implant’s surface; and (iii) a HF stimulation set-up able to promote matrix mineralization and ideal for monitoring osseointegration states. We here assessed the therapeutic potential of these cosurface electrodes powered by sensing-compatible HF excitations in pre-osteoblasts and human adipose-derived mesenchymal stem cells (hASCs).

## Results

### HF electrical stimuli delivered by the capacitive interdigitated system

**Fig. 1:**
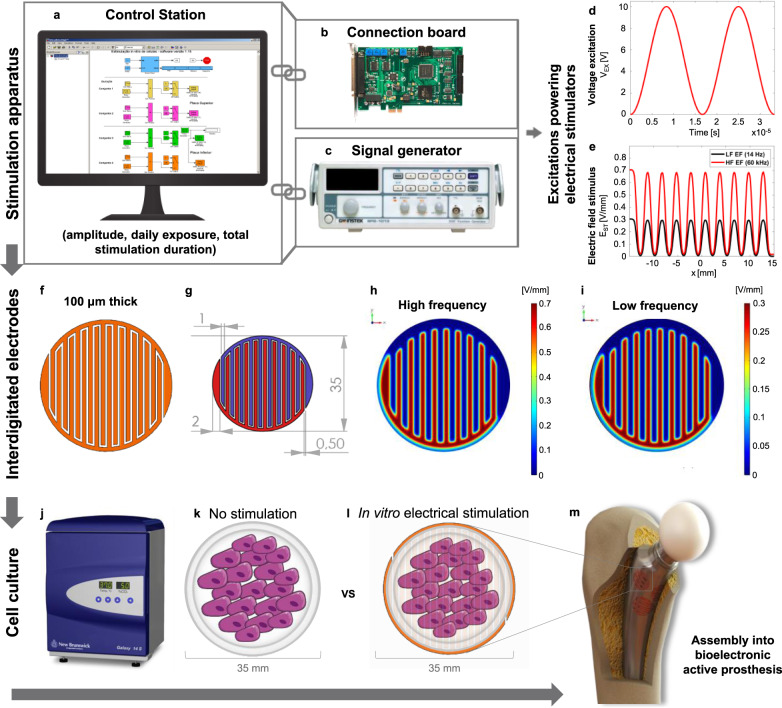
Outline of the capacitive stimulation device used for cells electrical stimulation. The stimulation apparatus comprises a control station (**a**) coupled to an I/O cardboard (**b**) for signal conditioning, and a signal generator (**c**) that ensures the generation of high-frequency (HF) excitations. The control station runs a real-time application that configures the excitation amplitude, daily exposure, and total stimulation duration. HF voltage excitations (60 kHz) have a defined waveform (**d**). Maximum electric field (EF) stimuli [0.7 vs 0.3 V/mm] are delivered for high- (60 kHz) or low-frequency (LF; 14 Hz) excitations (**e**), as predicted by a finite element computational model implemented using COMSOL Multiphysics (v. 5.3). The 100-μm-thick cosurface interdigitated electrodes (**f**) have a 35 mm diameter architecture (measures in mm in **g**). The electrode delivers frequency- and region-dependent EF stimuli to cells, with maximum EFs imposed over positively-charged electrodes and minimum EFs over the grounded electrodes. Stimuli distribution and strength along the *xy*-plane was determined under high- (**h**) and low-(**i**) frequency stimulation. In vitro stimulation was performed inside an instrumented CO_2_ incubator (**j**) connected to the stimulation apparatus, and cultures were grown in 35 mm culture dishes. The incubator contains both the control culture dishes (**k**) and the dishes receiving electrical stimulation, which are placed above each stimulator (**l**). These thin 35 mm electrodes are planned for assembly into bioelectronic active implants (**m**) to improve osseointegration around the bone–implant interface.

A capacitive interdigitated system was designed and tested when delivering HF electrical stimulation. Composed by control (Fig. 1a, b) and excitation (Fig. 1c) systems, and by capacitive interdigitated stimulators (Fig. 1f), this stimulation system is based on customized cosurface interdigitated electrodes to stimulate cells and/or target tissues. Interdigitated stimulators, made of copper to ensure high electrical conductivity, have an overall diameter of 35 mm and 100 µm thickness. A single stimulator comprises two interdigitated electrodes, each with ten strips fitting the intermediate spaces between the ten strips of the complementary electrode (Fig. 1g). Each electrode has 1-mm-wide stripes (outermost stripes with 2 mm width) and 0.5 mm gaps between electrodes. The stimulation system design will be ultimately defined considering implant geometry and personalized requirements, like the number of electrodes or the implant regions where actuation is needed (Fig. 1m).

Each stimulator (pair of interdigitated electrodes) was positioned over a polycarbonate substrate (Supplementary Fig. 1) and fixed on a tray placed inside an instrumented incubator (Fig. 1j). The polystyrene dishes (35 mm) containing the cell cultures were placed on top of each stimulator (experimental condition, Fig. 1l) or on an empty tray (control condition, Fig. 1k). This design ensures no direct contact between cells and electrodes, as expected in CC electrical stimulation where EFs are delivered to target tissues in a contactless manner. Polystyrene dishes and polycarbonate substrates were used given their high electrical resistivity. Based on a thorough data mining on CC stimulation osteogenic effects and on our pilot experiments^6^, each electrode was electrically powered by a 0–10 V sinusoidal voltage stimulus (*V*_EX_) (Fig. 1d), as expressed by equation (1), where *V*_pp_ is the 10 V peak-to-peak voltage and *f*_e_ is the 60 kHz HF excitation.

1 $$V_{\mathrm {EX}} = \frac{{V_{{\mathrm {pp}}}}}{2} + \frac{{V_{{\mathrm {pp}}}}}{2}{\mathrm {sign}}(\sin (2\pi f_{\mathrm e}t))$$

To predict the stimulus’ EF delivered to cells by the interdigitated stimulator, a numerical simulation was performed by a computational model developed using COMSOL Multiphysics (v.5.3, COMSOL). This model was engineered according to a set of structured domains already validated in silico and in vitro to predict electromagnetic stimuli delivered to cells during proliferation and differentiation stages^3,6,35^ (Supplementary Fig. 1). The stimuli dynamics (*E*_ST_) along the cellular tissue is sinusoidal (Fig. 1e), as defined by Eq. (2), where *E*_pp_ is around 0.7 V/mm peak-to-peak EF and *f*_e_ is the 60 kHz stimulation.

2 $$E_{{\mathrm {ST}}} = \frac{{E_{{\mathrm {pp}}}}}{2} + \frac{{E_{{\mathrm {pp}}}}}{2}{\mathrm {sign}}(\sin (2\pi f_{\mathrm e}t))$$

The stimuli dynamics was also determined for the LF excitation of 14 Hz and, although the same sinusoidal waveform was observed, the associated EF stimuli *E*_pp_ is only around 0.3 V/mm. The region-dependent EF was predicted along the cellular tissue. Under HF stimulation, the highest stimuli (0.7 V/mm) occur above the positively-charged electrodes, while the lowest stimuli (~0 V/mm) occur above the negatively-charged electrodes (Fig. 1e, h). Similar heterogeneity was found for LF stimulation (Fig. 1e, i) with highest (0.3 V/mm) and null stimuli occurring above the positively and negatively-charged electrodes, respectively.

### HF capacitive stimulator effects on osteoblasts proliferation and matrix maturation

Capacitive electrical stimulation of 0.7 V/mm and 60 kHz (defined by *E*_ST_), henceforth designated as *Stim* condition, was delivered for 30 min/day to MC3T3 pre-osteoblasts over 28 days in vitro (DIV). Cells’ metabolism and proliferation under *Stim* or *Ctrl* (no stimulation) conditions were determined by assessing metabolic activity, protein and DNA contents. Similar metabolic profiles were verified in both *Ctrl* and *Stim* conditions, growing about two-fold from baseline (1 DIV) to 5 DIV, and stabilizing onwards (Supplementary Fig. 2a). This initial period of increased metabolism is usually related to cellular proliferation^55,56^ but resazurin may have lost sensitivity at 7 DIV, since the cells appear to keep growing until 14 DIV, as revealed by total protein content profiles in Supplementary Fig. 2b. Again, no differences were observed between *Ctrl* and *Stim* populations. *Stim* seemed not to have significantly affected pre-osteoblasts early proliferation as occurs with other stimuli that accelerate maturation^3,34,57,58^. This was further confirmed by assessing the DNA content in the early 1–5 DIV proliferative period. No significant differences were found, and although *Stim* may have induced a certain initial delay in DNA content (3 DIV), this was recovered until 5 DIV (Supplementary Fig. 2c).

**Fig. 2:**
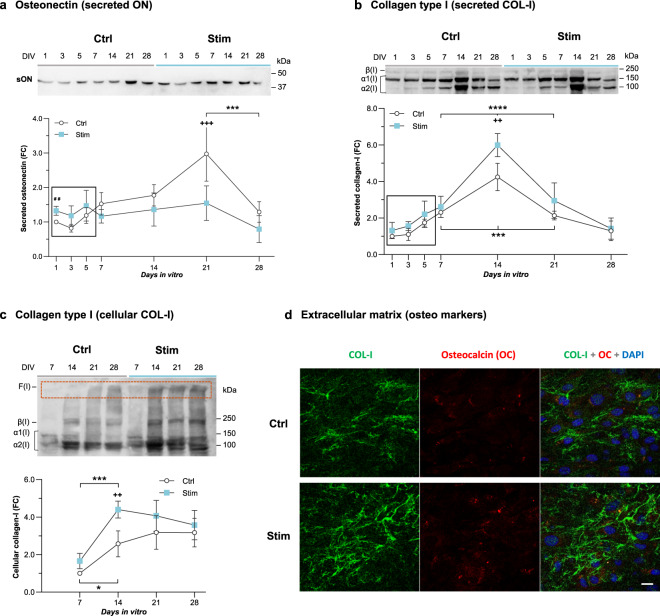
Matrix maturation of MC3T3 osteoblasts under high frequency stimulation. Immunoblot analyses and relative quantification of time-dependent profiles of **a** osteonectin (secreted ON, *n* = 4) and **b** collagen-I (secreted COL-I, *n* = 4) secreted into cells’ conditioned medium, and **c** intracellular collagen-I forms: α1(I) and α2(I) monomeric chains, β(I), dimeric forms and F(I), collagen fibrils (cellular COL-I; *n* = 4). Cellular COL-I image was filtered post-analysis (Image J “remove outliers” filter) for clearer presentation. Molecular weights are indicated to the right. DIV, days in vitro. Fold changes over *Ctrl* condition at first analytical time point. **d** Immunostaining of collagen-I (in green) and osteocalcin (in red) distributed in the extracellular matrix upon 28 DIV under *Ctrl* or *Stim* conditions. Cells’ nuclei were counterstained with DAPI (blue); scale bar, 20 μm. Results are presented as mean ± SD. Statistic symbols: (^+/#^) *Ctrl* vs *Stim* conditions; (*) between timepoints within the same condition; (^+/^*) two-way ANOVA; (^#^) one-way ANOVA; *P < 0.05, ^++/##^*P* < 0.01, ***^/+++^*P* < 0.001, ****P < 0.0001.

To analyze how daily *Stim* affected the matrix maturation phase, the production and secretion of relevant matrix proteins by osteoblasts were monitored. The secretion of osteonectin, a non-collagenous protein active during osteoblast maturation, appeared to be anticipated under electrical stimulation with our system. Osteonectin secretion under *Stim* conditions tended to be higher at earlier time points (1–5 DIV) (Fig. 2a), when compared to *Ctrl* conditions, and fairly remained steady until 21 DIV. Under *Ctrl* conditions, it raised with time and peaked at 21 DIV, equally dropping at 28 DIV for both conditions (Fig. 2a). Regarding collagen type-I, the most abundant extracellular matrix (ECM) protein, *Stim* also tended to increase its secretion during the early 1–5 DIV period, and induced a significant secretion peak at 14 DIV (Fig. 2b). More details on which collagen-I forms were increased by daily *Stim* were obtained by analyzing the cellular content (Fig. 2c). *Stim* generally increased cellular collagen-I over *Ctrl* throughout time, again more significantly at 14 DIV (Fig. 2c). This is the time point when cells stop to proliferate (Supplementary Fig. 2b) and onset major matrix maturation events^55^. Stimulation increased not only collagen-I α-monomeric chains and β-dimers but also high molecular weight γ-trimers and/or fibrils^55^ that barely entered the gel (Fig. 2c, “F(I)”). Immunocytochemistry (ICC) analyses confirmed enhanced deposition of collagen-I fibers on the ECM following 28 DIV under *Stim*, resulting in a tighter matrix (Fig. 2d, ICC of the ECM). Microphotographs also show extra deposition spots of another ECM-associated protein, osteocalcin, more recurrently found along the extracellular matrices of cells under *Stim*.

### Enhanced osteoblastic matrix mineralization upon HF stimulation

**Fig. 3:**
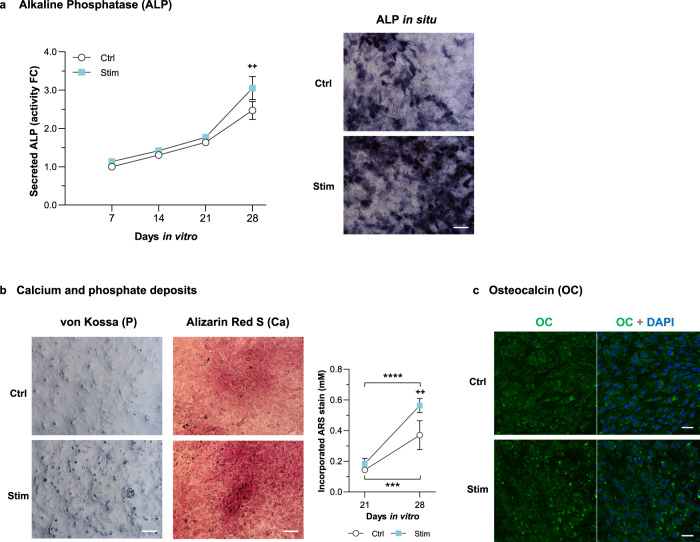
High frequency stimuli effects on ALP activity and mineralization of MC3T3 osteoblasts. **a** Activity profile of alkaline phosphatase (ALP) secreted to the cells' conditioned medium under *Ctrl* and *Stim* conditions over time (left graph, *n* = 3–4); right: in situ detection of ALP on cells cultured for 28 DIV under *Ctrl* and *Stim* conditions; scale bar, 100 μm. **b** Detection of calcium and phosphate deposits upon 28 DIV under *Ctrl* and *Stim* conditions by von Kossa staining of phosphate nodules and Alizarin Red S (ARS) staining of calcium-rich deposits; scale bars, 100 μm. Incorporated ARS stain was quantified at 21 and 28 DIV under *Ctrl* or *Stim* conditions (*n* = 3–4). **c** Immunostaining of osteocalcin (OC, in green) distributed in the cellular matrix upon 28 DIV under *Ctrl* or *Stim* conditions. Cells’ nuclei were counterstained with DAPI (blue); scale bar, 50 μm. Results are presented as mean ± SD. Statistic symbols: (^+^) *Ctrl* vs *Stim* conditions; (*) between timepoints within the same condition; ^++^*P* < 0.01, ***P < 0.001, ****P < 0.0001.

Osteoblastic differentiation culminates with an assembled mineralized matrix, which requires appropriate mineralization-associated proteins and enough mineral content. To determine how HF stimulation impacts osteoblasts’ mineralization, we assessed mineral deposits and protein markers. ALP synthesis was slightly but not significantly affected by *Stim* (Supplementary Fig. 3), but this condition increased ALP secretion. Enhanced extracellular ALP activity was observed for *Stim* at all time points tested, particularly at 28 DIV (Fig. 3a). An in situ ALP activity assay on the cells’ matrix confirmed increased enzymatic levels of this osteogenic marker upon 28 DIV of electrical stimulation (Fig. 3a “ALP in situ”, dark-blue spots).

Von Kossa and Alizarin Red S staining, targeting phosphate and calcium deposits, respectively, also consistently show enlarged deposition of both minerals under stimulation (Fig. 3b). Quantitative assessment confirmed that Alizarin stain was significantly more incorporated by cells’ extracellular matrices upon 28 DIV of HF *Stim* by our capacitive cosurface electrodes (Fig. 3b, right graph). ICC microphotographs again revealed increased osteocalcin deposition on the matrix of cells stimulated for 28 DIV (Fig. 3c).

### Influence of HF stimulation on secreted microvesicles

**Fig. 4:**
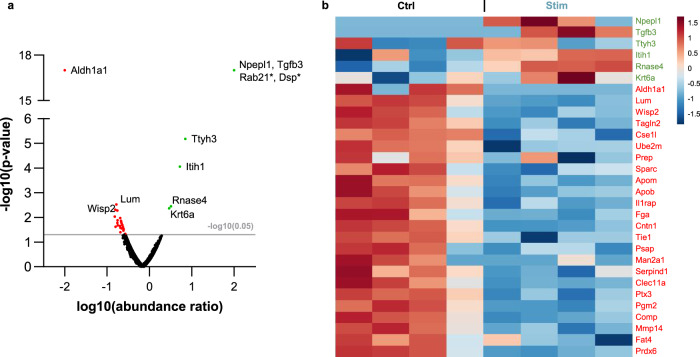
Proteomic analyses of osteoblast-secreted matrix microvesicles. **a** Volcano plot analysis of the 943 proteins identified by mass spectrometry. From these, 32 were significantly deregulated under *Stim* condition relatively to *Ctrl*: 8 proteins were considered significantly upregulated by HF stimulation, with abundance *Stim*/*Ctrl* ratios ≥2 (in green); 24 proteins were considered significantly downregulated by HF stimulation, with abundance *Stim*/*Ctrl* ratios ≤0.5 (in red; only the three top genes are identified in the graph). Data are plotted considering the log10 of the abundance *Stim/Ctrl* ratio and the −log10 of its *P* value (*n* = 4). The gray line at −log10 (0.05) represents the significance cut-off (*P* value = 0.05). Proteins marked with * were only detected in one sample from *Stim* condition. **b** Heatmap representing the relative abundance per sample of the 30 deregulated proteins detected in more than one sample per condition. Normalized abundances were log10 transformed and standardized for visualization of expression trends (*n* = 4). Proteins are sorted by the *P* value of their *Stim/Ctrl* ratio of relative abundances, and these values are color coded.

Osteoblast-secreted microvesicles (MVs) containing membranar ALP, ion metabolism-related enzymes, and phospholipids are suggested to function as mineralization nuclei when deposited into cells’ ECM^59^. Since electrical stimulation enhanced matrix mineralization, we further compared the proteomes of pooled MVs secreted by osteoblasts for 7–28 DIV under *Ctrl* or *Stim* conditions. Mass spectrometry analyses of the MVs pools identified 943 different proteins with two or more unique peptides and high confidence false discovery rate (FDR < 0.01). Proteins known to integrate MVs, such as AnnexinV, ENPP(1/2), EMILIN-1, PHOSPHO1, and ALP^59^ were found, as well as osteopontin and several collagen types (including I/III/V). ALP presented a non-significant 1.36-fold increase under *Stim* conditions. By volcano plot analysis, 32 proteins with abundance ratios ≥2 or ≤0.5 under *Stim* conditions, relatively to *Ctrl*, were considered significantly deregulated (Fig. 4a). The heatmap of 30 of these proteins, found in more than one of the biological quadruplicates per condition, is presented in Fig. 4b, where the proteins are sorted by *P* value of their abundance *Stim/Ctrl* ratios, and relative abundances values are color coded.

Although GO clustering is difficult to perform with low protein numbers, a functional enrichment analysis of the 32 deregulated proteins revealed some trends. Within the upregulated (UReg) group, “Extracellular matrix”, “collagen-containing extracellular matrix”, and “extracellular region” were the most enriched cellular component terms, while “extracellular space” and “extracellular region” were significantly enriched within the downregulated (DReg) group (Supplementary Fig. 4a, b). The UReg group was enriched in biological processes related to endosome transport, electrical conduction, and adhesion, whereas the DReg group was enriched in “chondrocyte proliferation”, “blood coagulation”, and “ossification” ones (Supplementary Fig. 4c, d). The UReg group was enriched in molecular functions related to chloride channels, transforming growth factor-beta (TGF-β) and “metalloexopeptidase activity”, while the DReg group was enriched in collagen and phospholipid binding and ECM constituent terms (Supplementary Fig. 4e, f). Functional hierarchy analysis via KEGG BRITE retrieved terms related to exosomes, signaling, ECM molecules, and hydrolases. Ensuing abstract mining revealed roles for these proteins in bone-related metabolism, suggesting that HF stimulation decreases cellular proliferation and some of the earlier maturation pathways, while promoting specific ECM maturation and mineralization ones (Supplementary Tables 1 and 2).

### Effects of HF stimulation on hASCs metabolism and osteodifferentiation

**Fig. 5:**
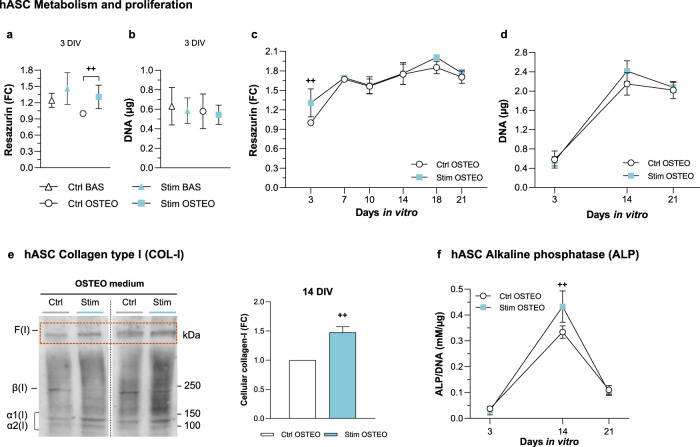
High frequency stimuli impacts on metabolism, proliferation, and osteogenic markers of human adipose-derived mesenchymal stem cells (hASCs). Early response of cells cultured for 3 DIV in BASAL or OSTEO medium and under *Ctrl* or *Stim* conditions, regarding **a** cellular metabolism (*n* = 9) and **b** DNA content (*n* = 3). Time-dependent profiles of cells’ **c** metabolic activity (*n* = 9–17) and **d** DNA content (*n* = 3–4), when cultured for 3–21 DIV in OSTEO medium and under *Ctrl* or *Stim* conditions. Fold changes were calculated over *Ctrl* OSTEO values at the initial time point (3 DIV). **e** Immunoblot analysis and relative quantification of cellular collagen-I (COL-I) levels. α1(I) and α2(I) monomeric chains, β(I), dimeric forms and F(I), collagen fibrils. Molecular weights are indicated to the right. DIV, days in vitro. Fold changes were calculated over *Ctrl* condition. **f** Alkaline phosphatase (ALP) quantification under *Ctrl* and *Stim* conditions (n = 3–4). Data were normalized to DNA content. Statistic symbols: (^+^) *Ctrl* vs *Stim* conditions; ^++^*P* < 0.01.

The osteogenic process involves both osteoblast precursors and mesenchymal stem cells (MSCs), the pre-osteoblasts precursor cells. Hence, the effects of HF stimuli delivered by these electrodes on hASCs differentiation were further assessed. Permittivity and conductivity parameters, similar to osteoblasts ones, were reported for hASCs stimulation using conductive scaffolds^60^ and for MSCs, respectively^61^. Comparison of hASCs growth on basal (BAS) or osteogenic (OSTEO) media and under *Stim* or *Ctrl* conditions was performed at the early 3 DIV time point. Higher metabolic activity was observed for *Stim* hASCs at 3 DIV, particularly significant for hASCs exposed to osteogenic medium (Fig. 5a, *Ctrl* OSTEO vs *Stim* OSTEO). This higher metabolism was not accompanied by increased proliferation, since *Stim* even slightly decreased the cells’ DNA content (Fig. 5b, *Stim* vs *Ctrl*), similarly to what occurs when osteogenesis is induced in *Ctrl* cells (Fig. 5b, *Ctrl* BAS vs *Ctrl* OSTEO). The growth characteristics of hASCs differentiating under OSTEO medium at subsequent time points (7–21 DIV) were relatively similar between *Stim* and *Ctrl* conditions, with cells proliferating until 14–18 DIV (Fig. 5c, d).

To characterize *Stim* influence on hASCs osteogenic differentiation, maturation and mineralization protein markers were assessed. HF stimulation again increased the cellular content of the maturation-associated collagen-I at 14 DIV (1.5-fold), also increasing the collagen-I fibers (Fig. 5e). Further, a significant surge in cellular ALP was observed also upon 14 DIV under *Stim* (Fig. 5f). Again, this is a time point where cells proliferation ends, and their differentiation program boosts.

### Capacitive HF stimulation impact on matrix mineralization of differentiating hASCs

**Fig. 6:**
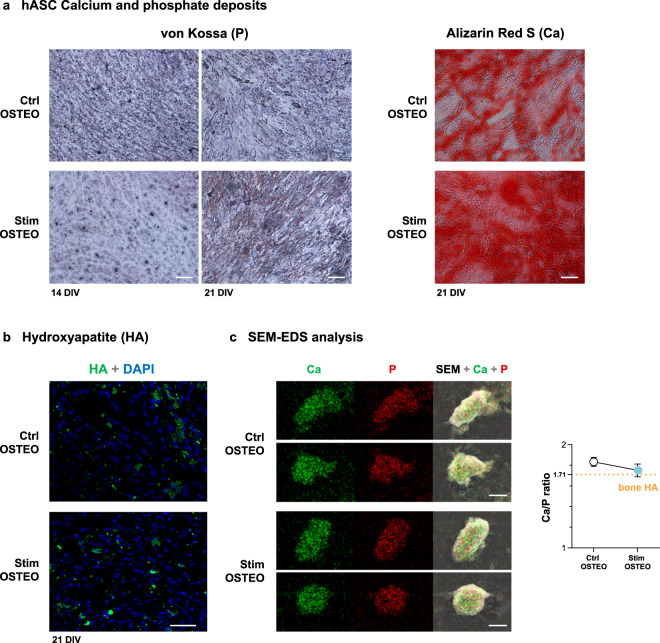
Effects of high frequency stimulation on hASCs matrix mineralization. **a** Detection of calcium and phosphate deposits under *Ctrl* and *Stim* conditions by von Kossa staining of phosphate nodules upon 14 and 21 DIV and Alizarin Red S (ARS) staining of calcium-rich deposits upon 21 DIV; scale bars, 50 μm. **b** Fluorescent staining of hydroxyapatite (HA, green) at 21 DIV. Nuclei were counterstained with DAPI (blue); scale bar, 100 μm. **c** Elemental analysis of hASCs extracellular matrices: SEM images and EDS elemental analysis by chemical mapping of calcium (Ca, green) and phosphorous (P, red) at the extracellular matrices of hASCs upon 21 DIV under *Ctrl* or *Stim* conditions; scale bar, 6 μm. The average calcium/phosphate ratios of these hydroxyapatite nodules were calculated using the atomic percentage of each element; the Ca/P ratio of 1.71 is the ratio of HA in natural bone (orange dashed line). Results are presented as mean ± SD (*n* = 4).

Effects of HF stimulation on MSCs mineralization was subsequently assessed through colorimetric and fluorescence assays upon 14–21 DIV of hASCs osteodifferentiating in OSTEO media and under daily HF *Stim*. Von Kossa staining revealed more phosphate-containing nodules in stimulated hASCs already at 14 DIV but particularly at 21 DIV (Fig. 6a “P”). In parallel, Alizarin Red S staining shows enhanced calcium-rich deposits on hASCs’ ECM at 21 DIV for cells under *Stim* (Fig. 6a “Ca”). To confirm these observations, hydroxyapatite (HA) nodules were labeled with a green fluorescence probe. Higher HA staining was observed for non-permeabilized hASCs under *Stim* for 21 DIV (Fig. 6b). Cellular permeabilization before fluorescence labeling revealed a green mantle spanning the osteo-like tissue, more stained and more continuous upon daily *Stim* delivery (Supplementary Fig. 5). Finally, to characterize the HA nodule-like structures in the hASC matrices at 21 DIV, SEM images were taken, revealing nodules’ morphologies, and their relative mineral composition was analyzed through Energy Dispersive X-Ray Spectroscopy (EDS/EDX) (Fig. 6c “Ca” and “P”; spectrum in Supplementary Fig. 6). Figure 6c images show that both calcium (green) and phosphorus (red) were evenly distributed across the nodules, and their overlapped chemical maps evidence co-localization of both elements across the structures (in yellow), for both conditions. Interestingly, nodules generated under *Stim* presented more defined shapes and regular borders, and less elements were observed loosely dispersed in the ECM (Fig. 6c). Further, HA nodules in hASCs under *Stim* conditions presented a Ca/P content ratio of 1.75 ± 0.07, highly similar to the one of HA nodules in natural bones (1.71 Ca/P ratio)^62^, while *Ctrl* hASCs presented a less-naturally occurring 1.83 ± 0.04 Ca/P ratio (Fig. 6c graph). These data strengthen previous results suggesting that *Stim* promotes more ordered formation of HA-like structures.

## Discussion

Bioelectronic implants designed so far are unable to control osseointegration or to deliver therapeutic stimuli according to physiological needs^3,6,23^. A possible solution may lean on capacitive architectures delivering HF stimuli to peri-implant tissues, with added potential of HF excitations in monitoring bone-implant integration states and assessing post-arthroplasty treatment evolution^23,36,63^. Our results demonstrate that daily HF stimulation neither disturbs metabolism nor proliferation of osteoblasts (Supplementary Fig. 2; only slight decrease unto 3 DIV) and promotes their maturation and matrix mineralization (Figs. 2–4). Besides, *Stim* increases the metabolism of early osteodifferentiating hASCs and may act synergistically with osteogenic media to initially attenuate hASCs proliferation at the onset of differentiation (Fig. 5a–d). Indeed, various cells decrease or stop proliferating when at the onset of differentiation^34,55,57,58,64–66^. HF stimulation delivered by cosurface electrodes may thus promote or accentuate hASCs pro-osteogenic commitment in basal and osteogenic media, respectively. Accordingly, electrical stimulation delivered by other apparatus was recently observed to induce long-lasting pro-osteogenic effects at early MSC osteodifferentiation phases^67^, and piezoelectrical stimulation induced MSCs differentiation into a 3D mineralized tissue^68^.

Once finished proliferating, pre-osteoblasts start differentiating and undergo ECM maturation. *Stim* appears to anticipate collagen-I and osteonectin secretions (Fig. 2a, b), what can actively impact earlier ECM assembly, given osteonectin role in Ca^2+^-binding, procollagen processing, and collagen fibril formation^69^. Also, *Stim* strongly increased collagen-I synthesis in osteoblasts, particularly at the matrix maturation phase (1.7-fold for cellular COL-I at 14 DIV, Fig. 2c), and promoted the formation of high molecular weight collagen-I fibrils (Fig. 2c). Altogether, this leads to an ECM with better organized fibril-like collagen-I structures, comparatively to *Ctrl* (Fig. 2d, *Stim*). Moreover, HF stimulation promoted extracellular ALP activity in osteoblasts from 7 DIV onwards, significantly higher at 28 DIV, when intensified cell-layer ALP activity is also observed (Fig. 3a). ALP, whose levels increase in maturing osteoblasts until differentiation into osteocytes^70^, is also a major contributor to matrix maturation, with intense ALP activity detected in bone cells’ membranes and ECM^59^. ALP is essential for mineralization, hydrolyzing inorganic pyrophosphates (that disturb mineralization) into PO4^3−^ monomers that, together with Ca^2+^, initiate crystalline nucleation^59^. Increased ALP levels thus contribute to augmented mineral deposition^71^, here confirmed by von Kossa and Alizarin matrix staining (Fig. 3b). Besides inorganic mineral content, the organic matrix comprises collagen-I and accessory proteins such as osteocalcin, a structure-directing molecule associated with collagen-I fibrils^72^. Indeed, both osteocalcin deposition (Fig. 3c) and its association with collagen-I fibrils (Fig. 2d) were promoted by HF stimulation. The HF stimulation impact on mineralization is reinforced considering the osteocalcin role in mediating HA crystals nucleation and growth^72^.

HF stimulation appears to promote mineralization via synthesis and particularly secretion of important structural and regulatory proteins. Considering the emerging reports of secreted MVs as mineralization nucleation initializers^59^, the differential protein content of extracellular MVs, secreted by osteoblasts under daily HF stimulation, was assessed. From the 1429 proteins identified, 943 had high confidence FDR (<0.01) and two or more unique peptides, including various MVs markers such as ALP. A subset of 32 proteins were considered significantly deregulated by volcano plot analysis (Fig. 4a, *P* values <0.05; *q* values = 0.0; heatmap in Fig. 4b). Included were eight upregulated MVs proteins related to different aspects of bone metabolism (osteodifferentiation, matrix stability, matrix mineralization) and voltage excitation (Supplementary Table 1), like **TGFB3**, a known osteogenic marker that is upregulated in bone regeneration, together with BMP and SMAD, other TGFB3 signaling proteins^73^. Accordingly, the *Stim* upregulated proteins were enriched in biological processes related to SMAD (Supplementary Fig. 4c). Also upregulated was **NPEPL1**, a predicted aminopeptidase, which is a group of enzymes upregulated during osteodifferentiation and believed to degrade matrix organic components to enable mineralization^74,75^. **TTYH3**, a Ca^2+^-activated chloride channel expressed in excitable tissues and whose gene deletion was clinically associated with low bone mineralization^76,77^, was also UReg. Proteins contributing to ECM stability and cellular adhesion were also found upregulated, namely **ITIH1**, from the ITI family of plasma protease inhibitors; **DSP**, one of the highest upregulated proteins during osteoblast differentiation; and **Rab21** that increases integrin-dependent cellular adhesion to collagen^78–80^, although DSP and Rab21 were found significantly upregulated by *Stim* but just in one sample (Fig. 4).

Associations to bone metabolism (confirmed or putative) were also found for the 24 downregulated MVs proteins, including roles in stemness; commitment into the osteo-lineage or other lineages; functions in earlier osteodifferentiation stages; bone growth inhibition; and/or osteoclastogenesis promotion. Examples of downregulated proteins upon *Stim* are contactin (**CNTN1**), which regulates TNFα in bone development^81^; osteolectin (**CLEC11A**), which promotes osteogenesis^82^ and is highly enriched in extracellular MSCs vesicles^83^; **PTX3**, an osteoblast differentiation promoter, highly expressed by precursor but not mature osteoblasts^84^; **TIE1**, a tyrosine-protein kinase receptor downregulated in neurotrophin-3-induced osteogenesis^85^; peroxiredoxin-6 (**PRDX6**), which inhibits osteogenic differentiation, impairing ALP activity and mineralized nodule formation^86^. Other downregulated proteins reported to decrease bone mass are **ALDH1A1**, **PGM1**, **SERPIND1**, and the collagenolytic metalloendopeptidase **MMP14**, all involved in RANKL-mediated osteoclast differentiation (Supplementary Table 2). The enzyme **ALDH1A1**, involved in PPARγ-mediated bone loss, is transcriptionally inhibited by the osteogenic Smad4^87^ and its deficiency induces BMP2 and increases bone mass in vivo^88^. Proteins with functions in ECM remodeling and bone mineralization were also found downregulated. This apparent contradiction may be attributed to *Stim* timing-related effects. Indeed, *Stim* may accelerate matrix maturation and mineralization and result in a global apparent downregulation of some proteins in the time interval analyzed (7–28 DIV). This is in accordance with the observed tendency to decrease proliferation in osteoblasts and hASCs, while promoting osteodifferentiating events (particularly secretion of ECM proteins) at earlier time points, thus accelerating osteodifferentiation through proteome shifts and protein trafficking. Aligned with this is the apparent DReg of **LUM**, a bone matrix component associated to collagen secretion/fibrillogenesis that is usually upregulated before the mineralization onset, returning to baseline levels thereafter^89^. **SPARC** (alias osteonectin) is also downregulated at 7–28 DIV, consistent with its decreased secretion at this interval (Fig. 2a). The secretion of this Ca^2+^- and HA-binding protein was actually anticipated by *Stim* to earlier differentiation days (Figs. 2a, 1–5 DIV), and the same may have occurred for other apparently downregulated proteins. Further, osteonectin ECM signal was not decreased at 21 DIV (Supplementary Fig. 7), and earlier osteonectin deposition on an emergent ECM would contribute to a more ordered and enhanced bone mineralization, as here observed. Regarding *Stim* osteogenic effects in differentiating hASCs, the uppermost ALP activity at 14 DIV (Fig. 5f) accurately correlates with increased round-shaped phosphate deposits stained by von Kossa (Fig. 6a, *Stim*). These initial crystalline nuclei grow and later elongate radially^59^, and von Kossa staining at 21 DIV revealed a high number of needle-shaped structures suggesting proper radial elongation under stimulation (Fig. 6a, *Stim*). Correspondingly, calcium-rich deposits and HA staining are increased at 21 DIV (Fig. 6a, b, *Stim*), undoubtedly confirming that mineralization is boosted by HF stimulation. SEM-EDS analyses of HA-like structures further revealed a more ordered HA formation in *Stim* hASCs, with nodules presenting higher structural organization and a Ca/P ratio closer to natural bone HA (Fig. 6c).

Our findings emphasize the potential translation of these capacitive stimulators into orthopedic implants, guaranteeing both monitoring of bone–implant interface and therapeutic electrostimulation for bone growth around the implant. An implant coated with a biocompatible material with electrical resistivity and instrumented with these electrodes below its surface is expected to perform very well for osseointegration after long-term implantation. The electrodes are designed to sense local loosening around specific implant regions, which will generate a warning signal to the clinician, who will activate those same, specific, electrodes at the loosening site to deliver therapeutic stimuli and promote osseointegration. The stimulators are placed below the implant’s surface, being highly improbable the formation of fibrous capsules that could locally lower stimulation efficiency at the fibrotic spots. Nevertheless, if any fibrous capsules are detected around the implant during pilot in vivo studies, anti-fibroblastic proliferation drugs may be used in combination^90^. To dismiss other clinical concerns, such as heat generation/accumulation on the biological tissues as a consequence of HF stimulation, a thermographic analysis was carried out to confirm that no changes in temperature occur after HF stimulation (Supplementary Fig. 8). This is as expected, since heat generation is not a problem when delivering EFs with a residual magnetic component, as the ones delivered by the capacitive system here presented.

**Fig. 7:**
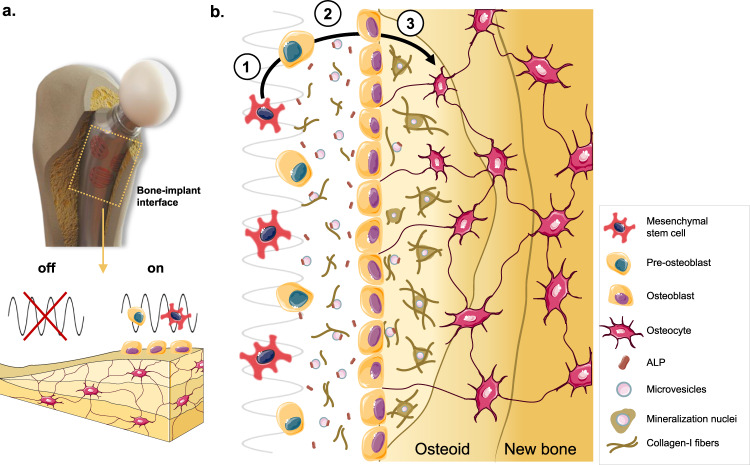
High osteogenic performance of cosurface capacitive stimulators at HF. Future innovative bioelectronic implants will deliver capacitive electrical stimulation of high frequency (HF, 60 kHz) around the bone–implant interface. HF stimuli promote osteodifferentiation of mesenchymal stem cells into pre-osteoblasts (1) and further proliferation and maturation of these into osteoblasts (2). HF stimulation induces active secretion of collagen-I, alkaline phosphatase (ALP), and microvesicles with membranar ALP and key matrix proteins. These microvesicles progress into mineralization nuclei for hydroxyapatite crystals early deposition, alongside with collagen-I fibers that are rearranged into a tight matrix. The mineralized matrix progressively entraps last-stage bone cells, the osteocytes (3) in the newly formed osteoid, that gradually becomes the new bone. Image elements taken from the Servier Medical ART free online vector image bank.

In conclusion, here we present an innovative capacitive electrostimulation delivery system of high osteoconductive and osteogenic performance, for flexible incorporation within future bioelectronic acting-sensing implants. This capacitive stimulator uses unique cosurface interdigitated electrodes, designed by us, operating at a HF to deliver customized stimuli to bone cells, that are ideal for future sensing/monitoring operations. This CC stimulation set-up is, to our knowledge, the first cosurface capacitive stimulation system with both osteoconductive and osteoinductive effects at both maturation and mineralization phases. Results reveal relevant stimulation-induced effects such as enhanced hASCs osteogenic differentiation, improved osteoconductive effects during osteoblastic maturation and specially, augmented and more ordered mineralization (summarized in Fig. 7). Overall, these effects most likely result from structural and regulatory osteogenic proteins that are differentially expressed and secreted under HF stimulation delivered by our capacitive system. To highlight, this work also includes, to our best knowledge, a first-time report on how electrical stimulation impacts the proteome of secreted MVs during osteodifferentiation and mineralization. The influence of stimulation on MSCs-secreted vesicles will next be pursued, given the impact of their cargoes (proteins, miRNAs) on osteodifferentiation pathways^91^. The cosurface delivery system here presented, operating at HF, is radically innovative given its: (a) dual osteoconductive and osteoinductive performance, (b) compatibility with monitoring/sensing operations, (c) flexible embeddability into implantable devices and adaptability to the implant’s surface topology, (d) ability to be incorporated in a capacitive network with a high number of electrodes that can (e) be independently controlled by clinicians, according to the osseointegration state monitored at each region of the implant/bone interface. These findings drive future exciting in vivo studies, using a multifunctional bioelectronic implant prototype instrumented with this capacitive electrical stimulation delivery system, aiming for a personalized HF acting-sensing therapeutic system of high fidelity and long duration.

## Methods

### Stimulation apparatus

The interdigitated stimulators were manufactured using printed circuit board (PCB) technology, which was achieved by etching the conductive pattern on cooper clad laminated FR-4 substrates. A real-time application was designed using Simulink (v. 7.3, Mathworks) and the Real Time Workshop (v. 7.3, Mathworks) to control the excitations powering the stimulators. It runs using the Real Time Windows Target (v. 3.3, Mathworks) kernel (Fig. 1a). Control signals were generated by an I/O card (MF 624, Humusoft) (Fig. 1b). The excitation signals were provided by a signal generator (SFG-1013, GW Instek) (Fig. 1c). Voltage excitations defined to power these cosurface stimulators were based on the ability of generated EF to stimulate osteodifferentiation in vitro (literature mining), with excitations up to 10 V being considered, since higher voltages can hardly be harvested by bioelectronic implants (constraints related to self-powering ability).

### Simulation details

The AC/DC module of COMSOL Multiphysics (v. 5.3, COMSOL) was used to develop all the computational models and to simulate the electrical stimuli delivered by the interdigitated stimulator. Six homogeneous and isotropic domains were included and modeled as before^3,6,35^ (Supplementary Fig. 1): (AI) air (41 mm diameter; 9.5 mm height); (SU) substrate (35 mm diameter; 0.5 mm thick); (EL) electrodes (1 mm stripes; 100 µm thick); (CD) culture dish (35 mm diameter; 0.5 mm thick; 2 mm height); (CT) cellular layer/tissue (34 mm diameter; 20 µm thick for cellular tissue); (CM) culture medium (34 mm diameter; 1 mm thick). These domains were tessellated by fine 3D meshes of second-order tetrahedral linear elements (Delaunay method). The mesh was refined for convergence errors lower than 2%. The homogeneous Neumann condition was defined to interior boundaries. The COMSOL physics interface “Magnetic and Electric Fields” was used to solve Maxwell’s equations in the frequency domain.

### Isolation, expansion, and characterization of human mesenchymal stem cells

Human adipose stem cells (hASCs) were isolated from subcutaneous adipose tissue obtained by liposuction, as before^92^. Tissues were obtained under cooperation with Hospital da Luz (Aveiro, Portugal), upon approval from the Hospital’s Clinical Ethics Committee (CEC) in November 2017. Informed consent was obtained, and all human samples were handled according to CEC approved guidelines. Briefly, adipose tissue aspirates were collected and kept at 4 °C in phosphate-buffered saline (PBS; Gibco, 10010023) containing 10% (v/v) antibiotics (penicillin/streptomycin; Gibco, 15140122). Samples were washed with PBS and digested using 0.05% (w/v) collagenase type II A (Sigma-Aldrich, C6885) for 45 min at 37 °C in a shaking water bath. Digested samples were filtered (200 µm) and centrifuged for 10 min at 800 × *g* and 4 °C. The stromal vascular fraction (SVF) obtained was resuspended in “basal” (BAS) medium: 2 mM l-glutamine-containing alpha-minimum essential medium (α-MEM; Gibco, 11900073), supplemented with 2.2 g/L sodium bicarbonate (Sigma-Aldrich, S5761), 10% (v/v) heat-inactivated fetal bovine serum (FBS; Gibco, 10270106), and 1% (v/v) antibiotic–antimycotic (10,000 units/mL penicillin; 10,000 μg/mL streptomycin, and 25 μg/mL Amphotericin B; Gibco, 15240062). Successful isolation of hASCs was confirmed by flow cytometry, used to assess the phenotypic expression of standard mesenchymal (98.1% of CD90-AlexaFluor647 and 91.8% of CD73-PE; BioLegend, 328116, 344004), hematopoietic (0.3% of CD34-FITC, BioLegend, 343604) and endothelial (0.1% of CD31-APC, BioLegend, 303116) markers (Supplementary Fig. 9). For that, 200,000 cells aliquots were harvested using TrypLE™ Express (Gibco, 12604013) at 37 °C for 5 min, and centrifuged. hASCs were resuspended in PBS with 2% (v/v) FBS, and the specified anti-CD antibodies diluted following the manufacturer instructions. After 1 h at RT, samples were washed with PBS, centrifuged, fixed in PBS with 1% (v/v) formaldehyde (Sigma-Aldrich, 252549), and analyzed in a flow cytometer (BD Accuri C6, CellQuest v3.3 software, BD Biosciences) equipped with FL1 (533/30 nm), FL2 (585/40 nm), and FL4 (675/25 nm) filters for emission detection.

### Mammalian cell’s in vitro stimulation assays

The mouse pre-osteoblastic MC3T3-E1 cells (CRL-2593, ATCC, Barcelona, Spain) were maintained in BAS medium, as before^3,6^. For in vitro stimulation, MC3T3-E1 cells were seeded on 35 mm dishes at 1 × 10^4^ cells/cm^2^ (~10% confluence) and BAS medium renewed every 3 days. hASCs, at passage 3, were also seeded on 35 mm dishes at 1 × 10^4^ cells/cm^2^ and cultured either in BAS medium or in osteogenic (OSTEO) medium: supplementation of BAS medium with 50 μg/mL ascorbic acid (l-ascorbic acid-2-phosphate, VWR, CAYM16457-5), 10 mM β-glycero0phosphate (Sigma-Aldrich, G9422), and 10 mM dexamethasone (Fisher Scientific, 10502631). High-frequency electrical stimulation in vitro (*Stim*, 60 kHz; 30 min/day) of MC3T3-E1 and hASCs was carried out in a CO_2_ instrumented incubator (Galaxy 14S, New Brunswick Scientific, Fig. 1j) equipped with the in-house developed capacitive stimulators^3,6^. Control samples (*Ctrl*) were maintained in parallel, in a different tray of the same incubator, without stimulation.

### Metabolic activity and cell proliferation (DNA content) assays

Metabolic activity was assessed at 1, 3, 5, 7, 14, 21, and 28 days in vitro (DIV) for MC3T3-E1 osteoblasts, and at 3, 7, 10, 14, 18, and 21 DIV for hASCs. Cells were incubated during 4 h with fresh complete medium containing 10% of a resazurin stock solution (0.1 mg/mL resazurin salt in PBS, Sigma-Aldrich, R7017). Resorufin absorbance was measured at 570 and 600 nm (Infinite 200 PRO, Tecan) and the ratio between optical densities (OD 570/OD 600) determined for each condition. The effects of delivering in vitro *Stim* by interdigitated electrodes, on cell metabolism, are presented as fold change over *Ctrl* levels at the first analytical time point of each assay. Quantification of total DNA was performed after cell lysis (Quant-iT™ PicoGreen® dsDNA assay kit, Life Technologies). MC3T3-E1 osteoblasts and hASCs were cultured under *Ctrl* or *Stim* conditions and harvested in 1–1.4 mL ultra-pure sterile water. After 1 h in a 37 °C shaking water bath, samples were frozen at −80 °C. Samples were defrosted and assessed according to the kit specifications. A standard curve for DNA analysis was generated with the provided dsDNA solution. After 10 min RT incubation, fluorescence was read in a microplate reader (Gen 5 2.01, Synergy HT, Biotek) with an excitation wavelength of 485/20 nm and emission of 528/20 nm.

### Immunoblot assays

Osteogenic differentiation markers were assessed in cells’ conditioned media and/or cellular lysates. Conditioned medium samples from MC3T3-E1 osteoblasts were collected at 1, 3, 5, 7, 14, 21, and 28 DIV with 10% sodium dodecyl sulfate (SDS) into 1% SDS solutions. MC3T3-E1 lysates at 7, 14, 21, and 28 DIV and hASCs lysates at 14 DIV were collected with 1% SDS. Protein content in lysates were quantified (Pierce^TM^ BCA Protein Assay Kit, Thermo Scientific, 23225). Mass-normalized cell lysates and resazurin-normalized conditioned medium samples were separated in 5–20% gradient SDS-polyacrylamide gels (SDS-PAGE) in Tris-Glycine buffer and further electrotransferred onto nitrocellulose membranes. Precision Plus Dual Color (Bio-Rad, 1610374) was used as protein standard. Membranes were first reversibly stained with Ponceau S (Sigma-Aldrich, P3504; 0.1% w/v in 5% acetic acid) and further used as loading controls^66^ (Supplementary Fig. 10). Primary antibodies, rabbit anti-collagen-I (1:2000; Novus Biologicals, NB600-408) and rabbit anti-osteonectin (1:1000; Novus Biologicals, NBP1-80972) were incubated with agitation, either ON at 4 °C or for 2 h at RT. Secondary antibodies HRP-linked anti-rabbit IgG (1:5000; GE Healthcare, NA934) were incubated for 1 h at RT with agitation. Proteins were detected through enhanced chemiluminescence (ECL) using Amersham ECL Select Western Blotting Detection Reagent (GE Healthcare, RPN2235) in a ChemiDoc Imaging System (Bio-Rad). Density analyses were performed with the Bio-Rad ImageLab Software and data were normalized to Ponceau S relative densities. *Ctrl* and *Stim* lysates were derived from a same experiment, run in the same gel, and density analysis of their protein bands in the respective blot was also performed in parallel.

### ICC and confocal microscopy

MC3T3-E1 osteoblasts grown on coverslips for 21 and 28 DIV in *Ctrl* and *Stim* conditions were fixed with 4% paraformaldehyde (PFA) for 15–20 min, and additionally subjected to antigen retrieval whenever necessary, by heating the coverslips at 95 °C for 10 min in 100 mM Tris solution pH 9.5 (NZYTech, MB01601) with 5% urea (w/v) (Sigma-Aldrich, U5378). Samples were further permeabilized with 0.2% Triton X-100/PBS and blocked with 0.2% BSA in PBS-T (0.1% Tween). The primary antibodies diluted in 0.2% BSA/PBS-T were then incubated for 2 h at RT: rabbit anti-collagen-I (1:500), rabbit anti-osteonectin (1:40), and mouse anti-osteocalcin (Novus Biologicals, H00000632-M01) (1:50). Secondary Alexa Fluor 488- and 594-conjugated antibodies (1:300) or Alexa Fluor 568 Phalloidin (Invitrogen, A12380; 1:50) were incubated for 1 h at RT. Coverslips were mounted with DAPI-containing Vectashield antifading reagent (Vector, H-1200) and images acquired using a Zeiss LSM 880 Airyscan confocal microscope (Zeiss, Germany).

### ALP activity assays

The activity of either secreted ALP or intracellular ALP in MC3T3-E1 osteoblasts was determined by enzymatic reaction, as before^3,6^. Medium samples were directly assessed. Cells were first permeabilized with 1% (v/v) Triton X-100 (Calbiochem, Merck, 9410) in PBS (30 min, 4 °C, 200 r.p.m.) and sonicated on ice. Homogenized samples were incubated for 1 h (37 °C, dark) with 200 µL ALP substrate [0.075% w/v ρ-nitrophenyl phosphate (Calbiochem, Merck, 4876) in an alkaline buffer solution (27.5 mM Na_2_CO_3_ (Sigma-Aldrich, 451614); 22.5 mM NaHCO_3_ (Sigma-Aldrich, S6014); 34 µM MgCl_2_ (Sigma-Aldrich, M8266); pH 10)]. The reaction was stopped with 0.02 M NaOH (Fine Chemicals, 30014-1000), and the solution’s optical density (OD) at 405 nm was further measured in a microplate reader (Tecan InfiniteM200). MC3T3-E1 ALP activity under stimulation is presented as fold change over the *Ctrl* activity levels at the initial timepoint. To detect ALP in situ activity in the mineralized matrix, MC3T3-E1 osteoblasts were grown for 28 DIV under *Ctrl* or *Stim* conditions, fixed with 4% PFA for 90 s (to avoid enzyme inactivation), and incubated for 15 min in the dark with an ALP reaction buffer [100 mM Tris (NZYTech, MB01601) at pH 9.0; 150 mM NaCl (NZYTech, MB15901), 1 mM MgCl_2_ (Sigma-Aldrich, M8266)] containing the ALP substrate 5-bromo-4-chloro-3-indolyl-phosphate (BCIP, 0.165 mg/mL) and the color coupler nitro blue tetrazolium (NBT, 0.33 mg/mL) (BCIP/NBT Promega, S3771). After a final wash with TBS-T, the blue stained spots were visible in the ECM and microphotographs of stained cell layers were taken with a Nikon Eclipse Ti-U microscope. ALP quantification in hASCs was performed as before^92^. Cells were harvested in ultra-pure water and aliquots incubated for 45 min (37 °C, dark) with 60 µL pNPP substrate [0.2% w/v 4-nitrophenyl-phosphate disodium salt hexahydrate (Sigma-Aldrich, 71768) in diethanolamine (1 M, Sigma-Aldrich, 31590)]. The reaction was stopped with 2 M NaOH and 0.2 mM EDTA (Sigma-Aldrich, E6758). A standard curve was prepared by diluting 4-nitrophenol solution (10 mM, Sigma-Aldrich, N7660) in the stop solution. The OD at 405 nm of the enzymatic products was read in a microplate reader (Synergy HTX, BioTek) and normalized to the cells’ DNA content.

### Alizarin Red S (ARS) calcium and von Kossa phosphate staining

ARS staining of calcium deposits was as defined by Gregory and Grady Gunn^93^. MC3T3-E1 osteoblasts grown for 21 and 28 DIV, and hASCs grown in OSTEO medium for 21 DIV, under *Ctrl* or *Stim* conditions, were fixed in 4% PFA for 15 min. Cells were incubated with a 40 mM ARS stock solution (pH 4.1) (Sigma-Aldrich, A5533) for 20 min at RT with agitation and further washed with distilled water. Microphotographs of stained cell layers were taken with a Nikon Eclipse Ti-U microscope. For quantification purposes, stained layers were further harvested in acetic acid (10% v/v) (Fisher Scientific, A/0400/PB17), the slurry was vortexed, heated at 85 °C, incubated on ice, and centrifuged at 20,000 × *g* for 15 min. Aliquots (500 μL) of the supernatant were neutralized with 200 μL ammonium hydroxide (10% v/v) (ACROS Organics, 390030010) and 150 μL triplicates plated in a 96-well plate, along with ARS standards (prepared from a 4 mM stock). Absorbance was read at 405 nm (Infinite 200 PRO) to determine the concentration of ARS dye incorporated by each sample.

The von Kossa^94^ protocol for calcium phosphate deposits was followed. MC3T3-E1 osteoblasts grown for 28 DIV and hASCs grown in OSTEO medium for 14 and 21 DIV, either in *Ctrl* or *Stim* conditions, were fixed in 4% PFA for 15 min. Cells were further incubated with 2% (w/v) aqueous silver nitrate solution (Sigma-Aldrich, S0139) under a UV light for 30 min, followed by incubation with 2.5% (w/v) aqueous sodium thiosulphate (Sigma-Aldrich, 72049) for 5 min to remove unreacted silver. Cells were washed with distilled water between solutions and ultimately stored in dry conditions at 4 °C. Calcium phosphate deposits, appearing as black nodules, were photographed in a Nikon Eclipse Ti-U microscope.

### Matrix MVs isolation and proteomic analyses

Conditioned media of MC3T3-E1 osteoblasts under *Ctrl* and *Stim* conditions, containing released matrix MVs, were collected at every medium change from 7 to 28 DIV and stored at −80 °C until MVs isolation. MVs were isolated from the medium by ultracentrifugation, based on refs. ^95,96^. Conditioned medium was first pre-cleared by two centrifugation steps: 300 × *g* at 25 °C for 5 min (5424, Eppendorf) and 18,500 × *g* at 4 °C for 25 min (5810 R, Eppendorf). The resulting supernatants were ultracentrifuged (Optima LE-80K, fixed-angle rotor 80Ti, Beckman Coulter GmbH, Krefeld, Germany) at 50,000 rpm for 60 min at 4 °C for pelleting the MVs. Upon ultracentrifugation, pellets were resuspended in 1% (v/v) protease inhibitors cocktail (Sigma-Aldrich, P8340) in PBS. Protein content was determined in each sample by the BCA assay (as above for cell lysates).

A comparative proteomic analysis between *Ctrl* and *Stim* samples was then performed, through nanoliquid chromatography coupled to tandem mass spectrometry (nanoLC-MS/MS), as before^97^. First, protein extracts (100 μg; *n* = 4) were reduced with tris-(2-carboxyethyl)-phosphine (TCEP), alkylated with chroloacetamide, and enzymatically digested using trypsin. Resulting tryptic peptides were then separated by liquid chromatography (nanoUHPLC) in an Ultimate 3000 system (Thermo Scientific) with a nanoelectrospray ionization (nanoESI) source coupled to a high-resolution accurate mass spectrometer (Q-Exactive Hybrid Quadrupole-Orbitrap, Thermo Scientific). Samples were loaded onto a trapping capillary column Acclaim PepMap C18 100 A˚ [3 µm particle size, 300 µm internal diameter (i.d.) × 5 mm, 160454, Thermo Scientific] and a mobile phase of 2% acetonitrile (ACN) and 0.1% formic acid (FA) at 10 μL/min. Upon 3 min loading, the trap column was switched to an EASY-Spray column [ES803A, PepMap RSLC, C18, 2 μm particle size, 75 µm i.d. × 50 cm, Thermo Scientific] at 300 nL/min. Sample elution was achieved by mixing solvent A (0.1% FA) and solvent B (80% ACN), using a linear gradient as follows: 5 min (2.5% B to 10% B), 120 min (10% B to 30% B), 20 min (30% B to 50% B), 5 min (50% B to 99% B), and 10 min (hold 99% B). The mass spectrometer was operated in the data-dependent (dd) acquisition mode. The analysis alternated between a full scan (*m/z* 380–1580) and a subsequent high-energy collisional dissociation (HCD) MS/MS of the 10 most intense peaks from full scan [normalized collision energy (NCE) 27%]. ESI spray voltage was 1.9 kV. Global settings were: use lock masses best (*m/z* 445.12003); lock mass injection Full MS; chrom. peak width (FWHM) 15 s. Full scan settings were: 70k resolution (*m/z* 200); automatic gain control (AGC) target 3e6; maximum injection time 120 ms. dd settings: minimum AGC target 8e3; intensity threshold 7.3e4; charge exclusion: unassigned, 1, 8, >8; peptide match preferred; exclude isotopes on; dynamic exclusion 45 s. MS2 settings were: microscans 1; 35k resolution (*m/z* 200); AGC target 2e5; maximum injection time 110 ms; isolation window 2.0 *m/z*; isolation offset 0.0 *m/z*; spectrum data type profile.

Raw data were further analyzed using the Proteome Discoverer 2.3.0.523 software (Thermo Scientific) by comparison against the *Mus musculus* proteome from the UniProt database (FASTA files obtained on May 2019). Tryptic peptides were identified by the Sequest HT search engine. The ion mass tolerance was defined at 10 ppm (for precursor ions) and 0.02 Da (for fragment ions). A maximum of two missing cleavage sites was allowed. Modifications were defined as follows: cysteine carbamidomethylation as constant modification; methionine oxidation and protein N-terminus acetylation as variable modifications. Peptide confidence settings were set as high. The following settings were applied to the processing node Percolator: maximum delta Cn 0.05; decoy database search target FDR 1%; validation based on *q*-value, which allows for more accurate determination of false positives for a given cut-off value, than using *P* value alone. Protein levels were determined through label-free quantification—LFQ methodologies. The mass spectrometry proteomics data have been deposited to the ProteomeXchange Consortium^98^ via the PRIDE^99^ partner repository with the dataset identifier PXD028551 and 10.6019/PXD028551.

Only proteins with a minimum of two unique peptides were considered for further analysis. The average abundances of proteins meeting the criteria above were compared between *Ctrl* and *Stim* conditions to determine the *Stim/Ctrl* abundance ratio. Through Volcano plot analysis (Fig. 4a), proteins whose *P* value <0.05 were assigned into two groups: upregulated (UReg) or downregulated (DReg) proteins under *Stim* conditions, considering *Stim*/*Ctrl* abundance ratios >2 or <0.5, respectively. Further hierachical clustering analysis of the deregulated proteins was performed using the MetaboAnalyst 5.0 online-available platform (metaboanalyst.ca) to obtain a graphical representation of the abundances (Fig. 4b). FunRich software v3.1.3^100,101^ was used to perform Gene ontology (GO) enrichment analyses, using the GO database and upon loading the appropriate *Mus musculus* database files (“House mouse” Taxon ID: 10090) (analyzed on Sept. 2019 and updated on April 2020). The ID map file “MOUSE_10090_idmapping.dat.gz” was downloaded from UniProt (ftp://ftp.uniprot.org/pub/databases/uniprot/current_release/knowledge base/idmapping/by_organism). The “mgi.gaf.gz” (i.e. gene association files from Mouse Genome Informatics) GO file was downloaded from http://current.geneontology.org/annotations/. Functional hierarchies were further analyzed through KEGG BRITE (genome.jp/kegg/brite.html) and an abstract mining for each deregulated protein was performed in *PubMed* (pubmed.ncbi.nlm.nih.gov) during Feb–Apr 2020, using the terms “bone”, “osteo”, “osseo”, “osteoblast”, “matrix”, and “mineralization”.

### HA fluorescent staining

hASCs cultured in OSTEO medium for 21 DIV, under *Ctrl* and *Stim* conditions, were fixed in 4% PFA for 15 min. The presence of HA crystals was assessed using the fluorescence OsteoImage™ Mineralization Assay kit (Lonza, PA-1503) according to the manufacturer’s instructions. Cells were stained with and without initial permeabilization by 0.1% Triton X-100 for 5 min at RT and incubated in Flash Phalloidin Red 594 (1:20 in PBS, Biolegend) for 30 min. Samples were counterstained with DAPI (1:1000 in PBS, 1 mg/mL, ThermoFisher Scientific) for 5 min at RT, and visualized by fluorescence microscopy (Axio Imager 2, Zeiss).

### Scanning electron microscopy and energy dispersive X-ray spectroscopy (SEM-EDS)

hASCs cultured in OSTEO medium for 21 DIV, under *Ctrl* and *Stim* conditions, were fixed in 4% PFA for 15 min and dehydrated in a graded series of ethanol. Following, samples were fixed with a carbon tape onto a graphite stub (Ted Pella) and sputtered by a thin film of carbon (K950X Turbo-Pumped Carbon Evaporator). Morphological and compositional analyses were carried out by scanning electron microscopy (SEM, SU-70, Hitachi, accelerating voltage 15 kV) coupled with an energy dispersive X-ray detector (EDS Bruker, Quantax 400 detector), as before^102^. Calcium (Ca) and phosphorous (P) peaks were determined by EDS spectra using Esprit software. EDS spectrum relative to the complete field of view of SEM-EDS images was obtained upon deconvolution of Ca and P peaks after background subtraction to calculate the Ca/P ratio of HA nodules (using % atom).

### Thermographic analyses

Thermographic images were acquired on three consecutive days, just before and immediately after applying the 30 min of 60 kHz electrical stimulation (*Stim*) to cells, using an Infrared Camera (Fluke Ti480). Images were taken inside and outside the incubator, these last for temperature assessment of isolated cultured dishes, and temperature measurements of culture dishes were averaged.

### Statistical analyses

Except when indicated otherwise, fold changes (FC) were calculated by comparing the raw data of each analyzed timepoint to the levels of the first control timepoint analyzed in each assay (taken as 1.0) and averaged between independent replicas. Quantification of DNA, protein content, incorporated ARS dye and ALP from hASCs were obtained using appropriate calibration curves. hASCs ALP values were further normalized to DNA content. All data are presented as mean ± SD of different biological replicas (*n*, indicated in each figure). Statistical analyses were conducted using the GraphPad Prism 8.0.1 software; confidence intervals of 95% were considered. Variance analysis was performed using two-way ANOVA and Tukey’s multiple comparisons test (for comparison between conditions considering time-dependent profiles). Variance analysis between conditions at the same time point was performed using one-way ANOVA and Tukey’s test (in Fig. 2a 1 DIV, Fig. 5a 3 DIV, Fig. 5e 14 DIV).

### Reporting summary

Further information on research design is available in the Nature Research Reporting Summary linked to this article.

### Supplementary information

Supplementary Information

Reporting Summary

## Data Availability

All relevant data will be made available by the authors upon request. The proteomic dataset generated during the study is available via the ProteomeXchange Consortium with the dataset identifier PXD028551 and 10.6019/PXD028551. All datasets used for Gene Ontology enrichment analysis are detailed in the “Methods” section.
