# Author Correction: Capacitive interdigitated system of high osteoinductive/conductive performance for personalized acting-sensing implants

**DOI:** 10.1038/s41536-024-00350-6

**Published:** 2024-01-20

**Authors:** Bárbara M. de Sousa, Clara R. Correia, Jorge A. F. Ferreira, João F. Mano, Edward P. Furlani, Marco P. Soares dos Santos, Sandra I. Vieira

**Affiliations:** 1 Department of Medical Sciences, Institute of Biomedicine (iBiMED), University of Aveiro, 3810-193 Aveiro, Portugal; 2 Department of Chemistry, CICECO – Aveiro Institute of Materials, University of Aveiro, 3810-193 Aveiro, Portugal; 3 Department of Mechanical Engineering, Centre for Mechanical Technology & Automation (TEMA), University of Aveiro, 3810-193 Aveiro, Portugal; 4 Department of Chemical and Biological Engineering, Department of Electrical Engineering, University at Buffalo (SUNY), Buffalo, NY 14260 USA; 5 Faculty of Engineering, Associated Laboratory for Energy, Transports and Aeronautics (LAETA), University of Porto, 4200-465 Porto, Portugal

Correction to: *npj Regenerative Medicine* 10.1038/s41536-021-00184-6, published online 23 November 2021

In the Acknowledgements section, the funding source ‘support to the Centre for Mechanical Technology & Automation (TEMA; UID/EMS/00481/2019) and to the Institute of Biomedicine (iBiMED; UID/BIM/04501/2019)’ should have read ‘support to the Centre for Mechanical Technology & Automation (TEMA, UIDB/00481/2020 and UIDP/00481/2020), and to the Institute of Biomedicine (iBiMED, UIDB/04501/2020 and UIDP/04501/2020)’. The original article has been corrected.

