# Supplementary material for: Capacitive interdigitated system of high osteoinductive/conductive performance for personalized acting-sensing implants
Source: NPJ Regen Med. 2021 Nov 23;6:80. doi: 10.1038/s41536-021-00184-6 (PMC8611088; doi:10.1038/s41536-021-00184-6)
Supplement: Supplementary file 1 — Supplementary Information [file 41536_2021_184_MOESM1_ESM.pdf]

Supplementary Information for

Capacitive interdigitated system of high osteoconductive/inductive performance for personalized acting-sensing implants

Bárbara M. de Sousa[1], Clara R. Correia[2], Jorge A. F. Ferreira[3], João F. Mano[2], Edward P. Furlani[4], Marco P. Soares dos Santos[3,5,#,*], Sandra I. Vieira[1,#,*]

[1)] Department of Medical Sciences, Institute of Biomedicine (iBiMED), University of Aveiro, 3810-193 Aveiro, Portugal.

[2)] Department of Chemistry, CICECO - Aveiro Institute of Materials, University of Aveiro, 3810-193 Aveiro, Portugal.

[3)] Department of Mechanical Engineering, Centre for Mechanical Technology & Automation (TEMA), University of Aveiro, 3810-193 Aveiro, Portugal.

[4)] Department of Chemical and Biological Engineering, Department of Electrical Engineering, University at Buffalo (SUNY), Buffalo, NY 14260, USA.

[5)] Faculty of Engineering, Associated Laboratory for Energy, Transports and Aeronautics (LAETA), University of Porto, 4200-465 Porto, Portugal.

# These authors have jointly supervised the work

* Corresponding authors: Sandra I. Vieira (biomedicine; sivieira@ua.pt, +351234247256); Marco Santos (bioengineering; marco.santos@ua.pt, +351234370830)

Running title: New highly osteodifferentiating capacitive active stimulator

Supplementary Figures 1 to 10

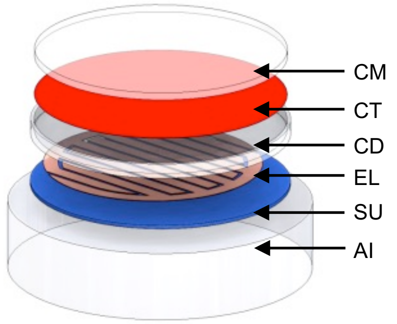

Supplementary Fig. 1 | The six homogeneous and isotropic domains used for computational modelling in COMSOL Multiphysics. (AI) air (41 mm diameter; 9.5 mm height); **(SU)** substrate (35 mm diameter; 0.5 mm thick); **(EL)** electrodes (1 mm stripes; 100 µm thick); **(CD)** culture dish (35 mm diameter; 0.5 mm thick; 2 mm height); **(CT)** cellular tissue (34 mm diameter; 20 µm thick for cellular tissue); **(CM)** culture medium (34 mm diameter; 1 mm thick).

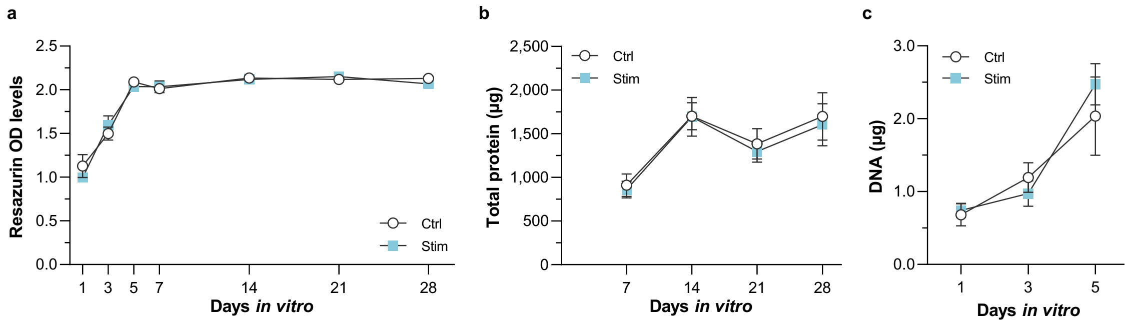

Supplementary Fig. 2 | Metabolism and proliferation of MC3T3 osteoblasts upon high frequency stimulation. a, Metabolic activity profile of cells under *Ctrl* and *Stim* conditions over time, obtained by the resazurin-based assay (n=3-6). **b,** Total protein content in cell lysates under *Ctrl* and *Stim* conditions over time, determined by the BCA assay *(*n=5). **c,** Cellular proliferation profile under *Ctrl* and *Stim* conditions from 1 to 5 DIV, determined by DNA content quantification (n=4). Results are presented as mean ± SD.

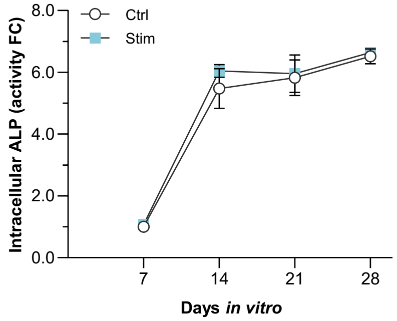

Supplementary Fig. 3 | Time-dependent profile of intracellular ALP activity under *Ctrl* and *Stim* conditions. Fold changes were calculated over *Ctrl* values at first time point, and results are presented as mean ± SD (n=3-4).

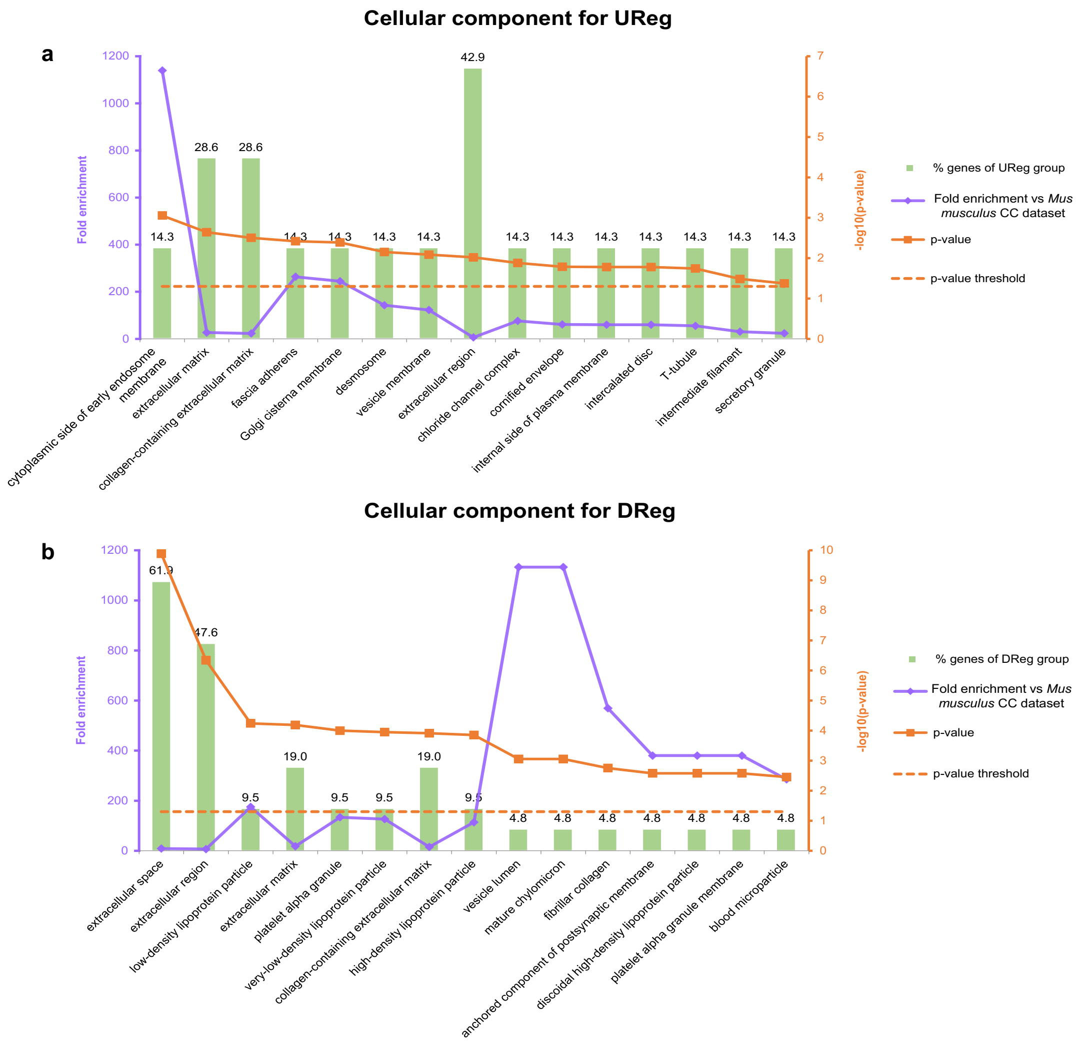

Supplementary Fig. 4 | Gene ontology enrichment analyses of differentially abundant proteins in microvesicles secreted by MC3T3 osteoblasts under high frequency (HF) daily stimulation for 28 days (7-28 days *in vitro*). Graphics with significantly altered GO terms were obtained upon GO enrichment analyses using the FunRich software, and comprise: the percentage of MV deregulated proteins with a specific GO term associated (green bars); the -Log10 of the p value for that percentage against the percentage of all proteins from the *Mus musculus* proteome associated to that specific GO term (orange markers and continuous line); the -Log10 of the p value = 0.05, indicating the significance cut-off (orange dashed line; to compare with orange continuous line); the fold enrichment of the GO term in each deregulated proteins dataset compared to the enrichment of the same GO term in the *Mus musculus* dataset (purple markers and line). **a, b,** Enrichment analysis of cellular components (CC) GO terms for proteins upregulated (UReg) and downregulated (DReg) by HF stimulation, respectively. **c, d,** Enrichment analysis of biological processes (BP) GO terms for MV proteins UReg and DReg by HF stimulation, respectively. **e, f,** Enrichment analysis of molecular functions (MF) GO terms for MV proteins UReg and DReg by HF stimulation, respectively. *(continues in following pages)*

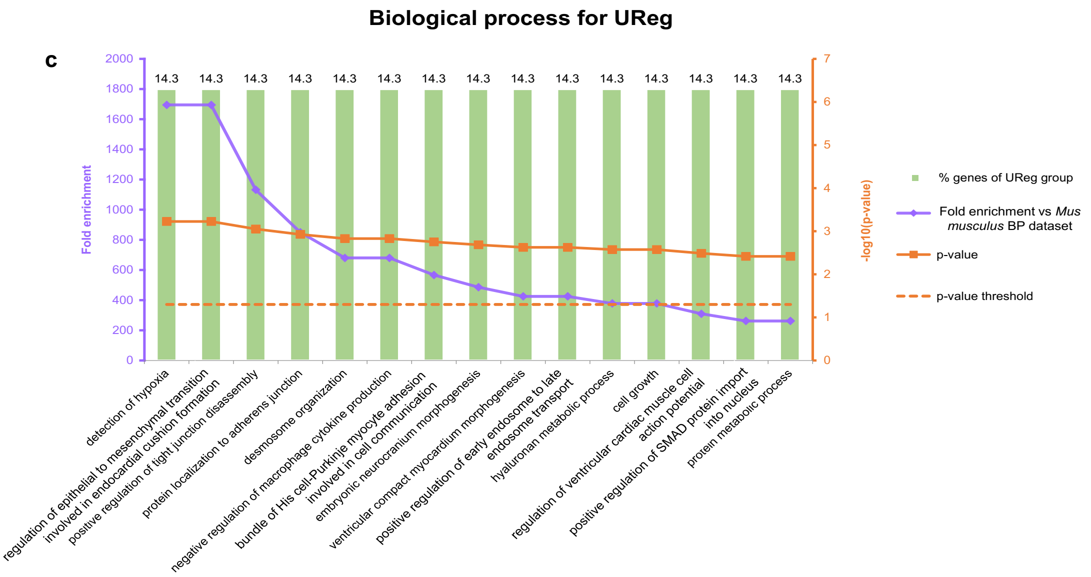

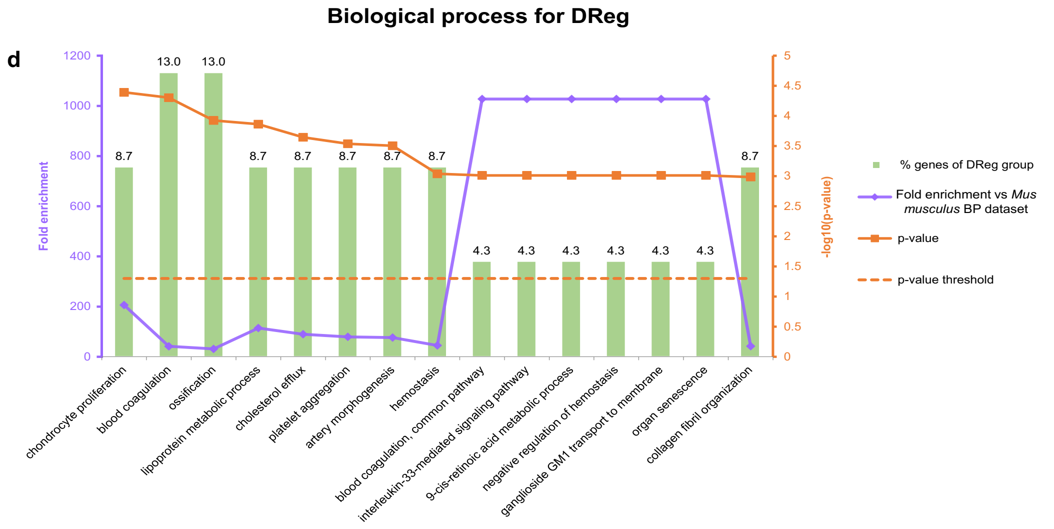

Supplementary Fig. 4 (cont.) **| Gene ontology enrichment analyses of differentially abundant proteins in microvesicles secreted by MC3T3 osteoblasts under high frequency (HF) daily stimulation for 28 days (7-28 days *in vitro*).**

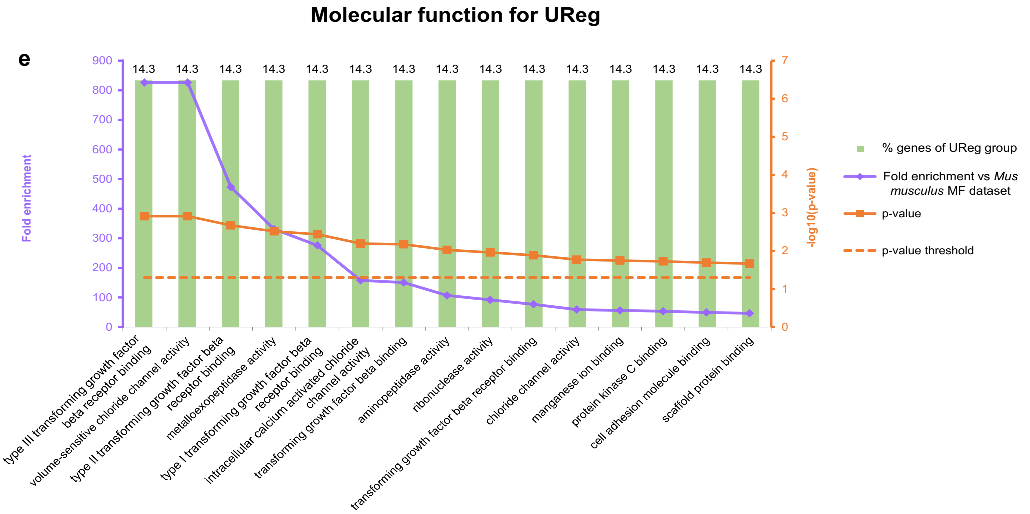

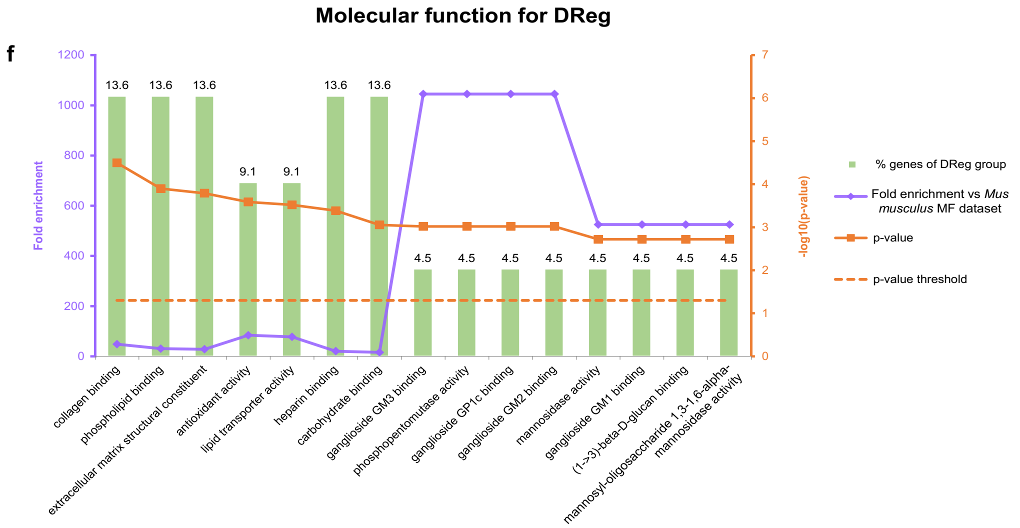

Supplementary Fig. 4 (cont.) **| Gene ontology enrichment analyses of differentially abundant proteins in microvesicles secreted by MC3T3 osteoblasts under high frequency (HF) daily stimulation for 28 days (7-28 days *in vitro*).**

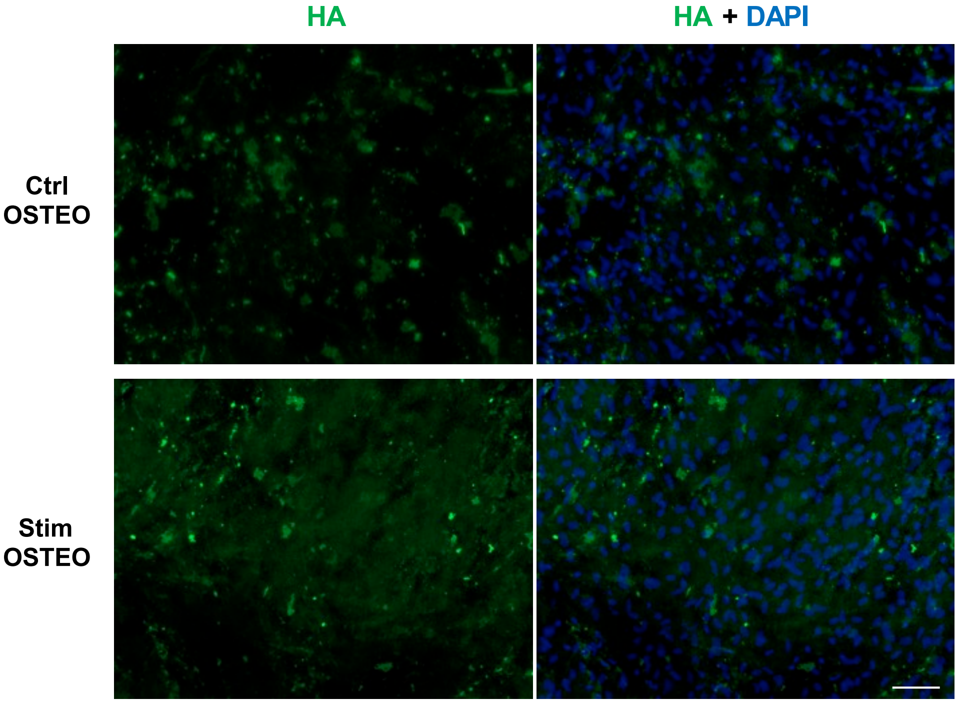

Supplementary Fig. 5 | Hydroxyapatite imaging in osteodifferentiating hASCs at 21 DIV, following cells pre-permeabilization. Hydroxyapatite (HA) in hASC cells cultured in OSTEO medium for 21 DIV was labelled with a green fluorescing dye (OsteoImage^{TM}). HA staining was performed in pre-permeabilized cells. Nuclei were counterstained with DAPI (in blue). Micrographs were taken with the same exposure time, and images' brightness and contrast equally optimized. Scale bar, 100 μm.

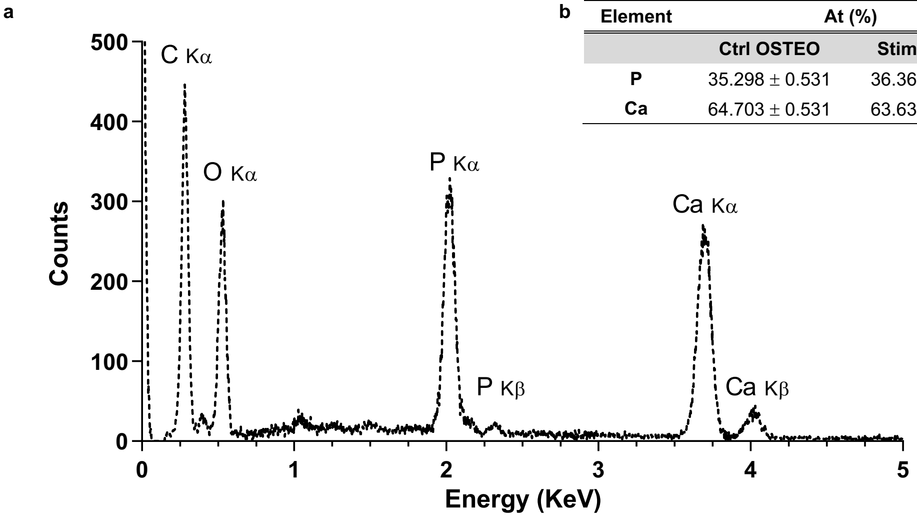

b

Element	At (%)	
	Ctrl OSTEO	Stim OSTEO
**P**	35.298 ± 0.531	36.363 ± 0.805
**Ca**	64.703 ± 0.531	63.638 ± 0.805

Supplementary Fig. 6 | EDS analysis of HA nodules in hASC matrices. a, EDS representative spectrum, presented as a graph plotting energy (KeV) versus the relative counts of the detected X-rays emitted by the sample. Peaks labelled as Kα and Kβ refer to electron transitions from outer to inner electron shells which emit radiation with different energies, corresponding to transitions from L to K shell and transitions from M to K shell, respectively. **b,** Atomic percentages of both Ca and P in HA nodules of hASC under *Ctr* and *Stim* conditions.

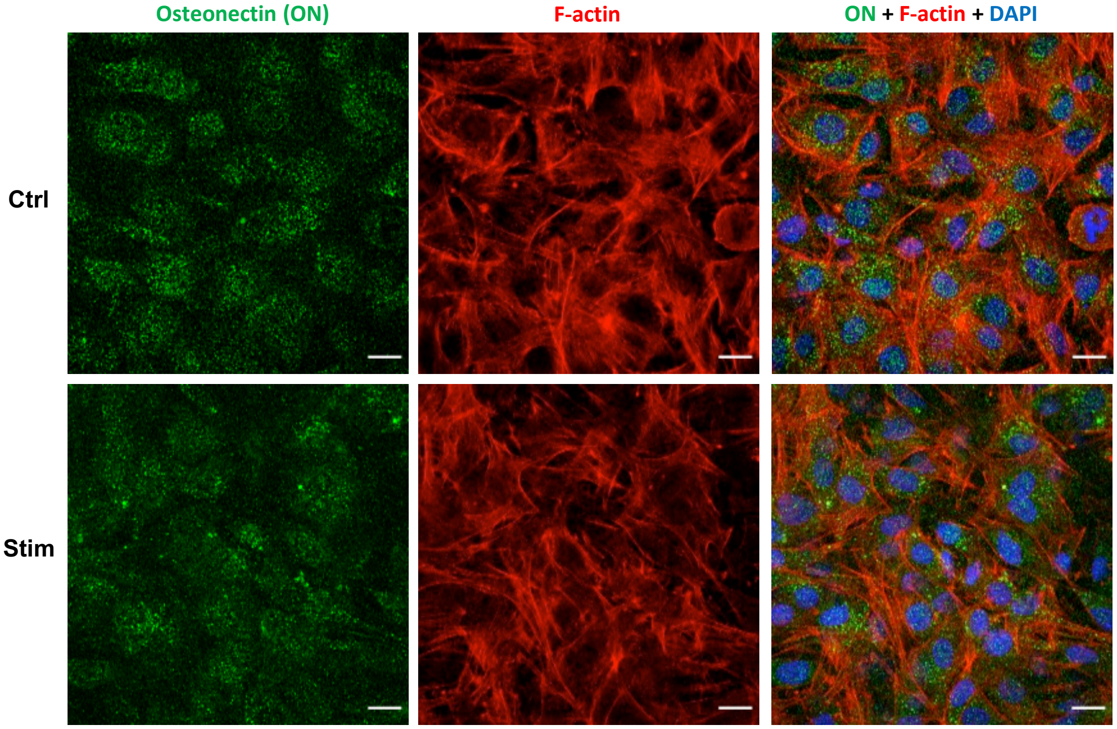

Supplementary Fig. 7 | Immunocytochemistry analysis of osteonectin distribution inside osteoblasts and throughout their extracellular matrix at 21 DIV. Osteonectin (ON) immunostaining (in green) and F-actin labelled with TexasRed-conjugated phalloidin (in red), in MC3T3 osteoblasts grown for 21 DIV under *Ctrl* or *Stim* conditions. Cells' nuclei were counterstained with DAPI (blue). Scale bar, 20 µm.

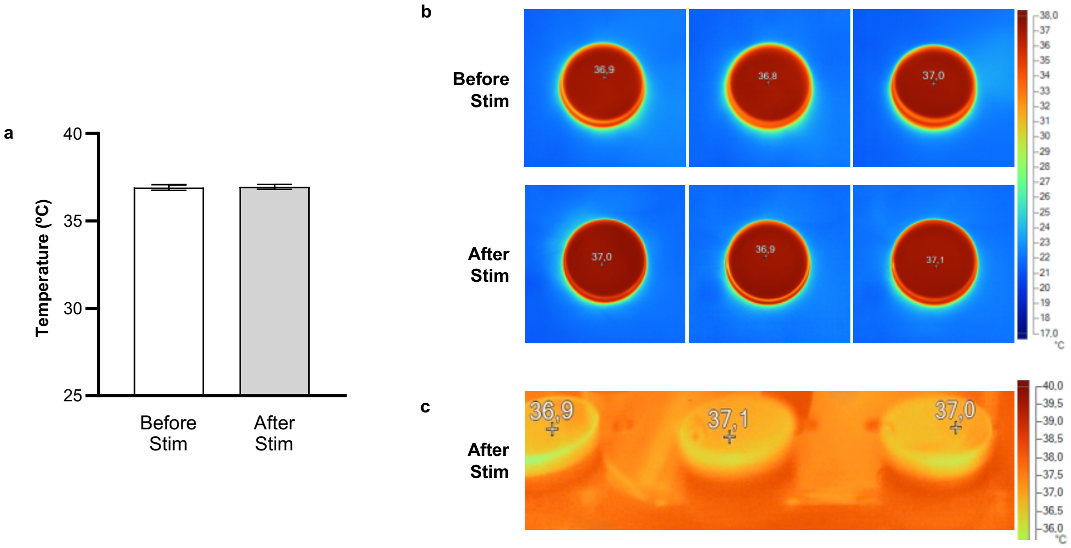

Supplementary Fig. 8 | Thermographic imaging assays before and after cells electrical stimulation. a. Average temperature of culture dishes before and after 30 min of 60 kHz electrical stimulation (*Stim*). Temperature measurements were taken on 3 consecutive days *in vitro* and presented as mean±SD (n=4). **b.** Illustrative examples of thermographic images taken outside the incubator for temperature assessment of isolated cultured dishes before and immediately after a 30 min *Stim* period. **c.** Illustrative example of a thermographic image taken inside the incubator after the same 30 min *Stim* period.

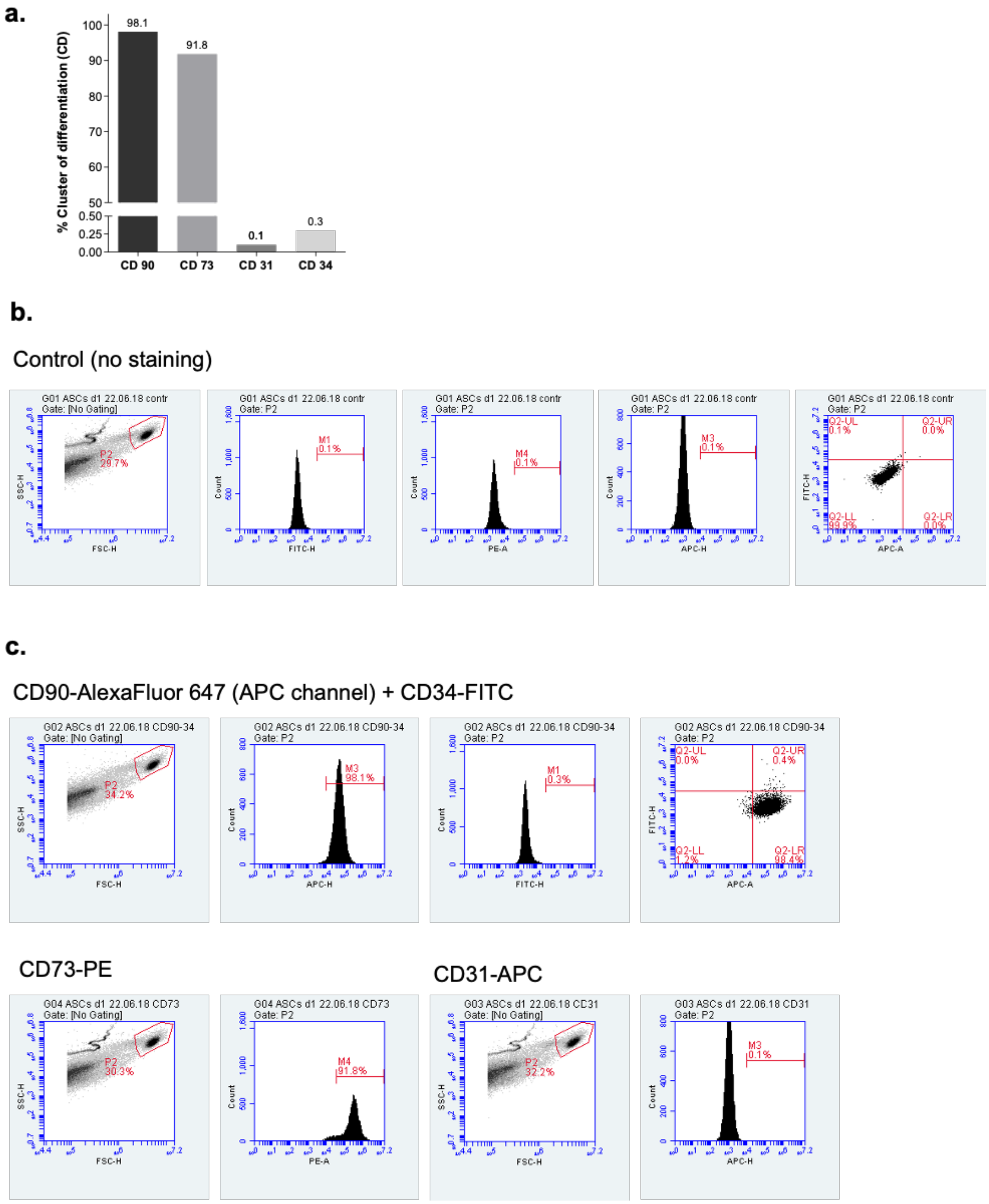

Supplementary Fig. 9 | Flow Cytometry Analysis of hASCs. a. Graphical representation of hASCs phenotypic expression of standard mesenchymal (CD90-AlexaFluor647 and CD73-PE), hematopoietic (CD34-FITC) and endothelial (CD31-APC) markers, to confirm the successful isolation of hASCs (% of gated relevant events, corresponding to c.a. 10,000 cells in total). **b.** Flow cytometry analysis of control unstained isolated cells (c.a. 30,000 events, with c.a. 10,000 cells) and gating strategies for exclusion of artifacts like tissue debris, based on size and complexity, by using the forward (FSC) and side (SSC) scatter dot plot (left), and autofluorescence in the FITC, PE, APC channels (fluorescence histograms and dot plot, at the right). **c.** Flow cytometry analyses of isolated cells stained with the mesenchymal CD90-AlexaFluor647 and the hematopoietic CD34-FITC markers, or separately stained with the mesenchymal CD73-PE and the endothelial CD31-APC markers. Left: SSC vs FSC dot plots of total events, and the relevant cellular population ('P2') further analysed; right: fluorescence (FITC, PE, APC) histograms of the respective CD stained P2 population.

a. MC3T3 pre-osteoblasts cellular lysates

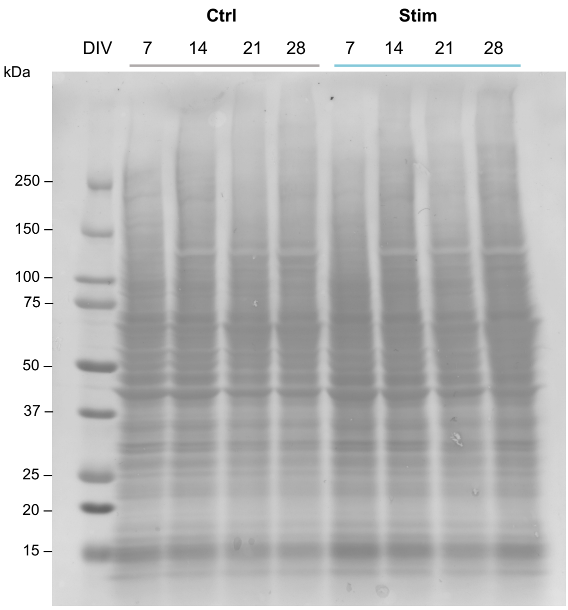

b. hASCs cellular lysates (OSTEO medium)

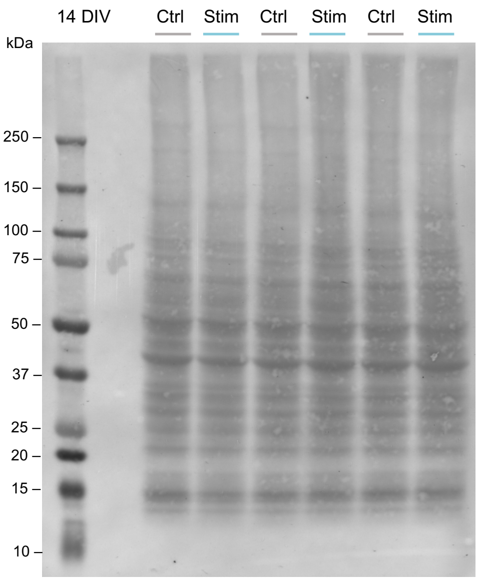

c. MC3T3 pre-osteoblasts media lysates

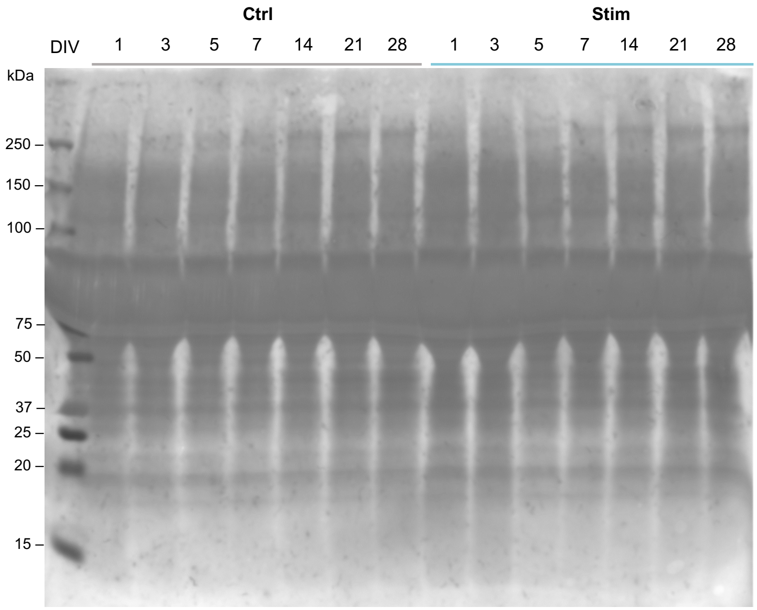

Supplementary Fig. 10 | Ponceau S staining of nitrocellulose blots for protein loading control. a, b, Ponceau S stained nitrocellulose membranes, used to control SDS-PAGE protein sample loading of MC3T3 cellular (Fig. **2c**) and hASCs (Fig. **5e**) lysates, respectively. Relative densitometries of lanes/samples were used to normalize immunoblot quantitative results of cellular collagen-I and osteonectin (MC3T3 cells) and collagen-I (hASCs). **c,** Ponceau S stain of nitrocellulose membranes, used to control SDS-PAGE sample loading of MC3T3 cells' conditioned media (Figs. **2a,b**). Ponceau S stain was used to assess a high degree of similarity in gel loading. Molecular weight indicated to the left. DIV, days *in vitro*.

Supplementary Tables 1 & 2 and Supplementary Discussion

Proteomic analysis of osteoblast-secreted microvesicles from 7 to 28 DIV

Microvesicles (MVs) secreted by maturing osteoblasts and deposited on their matrices are potential initializers of calcium phosphate mineral nucleation[1,2]. The effects of 30 min daily high frequency (HF) electrical stimulation (*Stim*) delivered by interdigitated capacitive electrodes on the mineralization nuclei were assessed by mass spectrometry in comparison to control condition without stimuli (*Ctrl*). The proteomic analysis was performed using a pool of conditioned media secreted by MC3T3-E1 cells from 7 to 28 days *in vitro* (DIV), to further evaluate the protein content of these extracellular MVs. From the 1429 different identified proteins with q-values < 0.05, 943 had 2 or more unique peptides (all with high confidence False Discovery Rates, FDR < 0.01); from these, 32 proteins had *Stim*/*Ctrl* abundances > 2 (upregulated, **UReg – Supplementary Table 1**) or < 0.5 downregulated (**DReg – Supplementary Table 2**) and were considered significantly deregulated by the Volcano plot software (p-values < 0.05; and q-values = 0.0).

Supplementary Table 1 | Microvesicles' proteins upregulated by daily high frequency stimulation. All proteins have high FDR confidence (<1%) and q-value = 0.0, and their levels considered significantly upregulated by the Volcano plot software; proteins concomitantly presenting adjusted p-value < 0.05 are shadowed (n=4).
AN, Uniprot accession number; GS, Gene Symbol; STIM / CTRL Abundance ratio, ratio of the relative protein abundance in *Stim* against *Ctrl* conditions. Abundance ratios of 100: proteins only detected in *Stim* conditions and not in *Ctr*; (*) proteins only detected in one *Stim* condition sample. Functional hierarchies' classification retrieved from KEGG BRITE (genome.jp/kegg/brite.html). Possible roles determined through abstract mining in *PubMed* (pubmed.ncbi.nlm.nih.gov), using the terms 'bone', 'osteo', 'osseo', 'osteoblast', 'matrix' and 'mineralization'.

AN - Protein name and GS	STIM / CTRL Abundance ratio	Functional hierarchies	Possible role in bone metabolism or known involvement in bone cells' proliferation or differentiation
Q6NSR8 - Probable aminopeptidase NPEPL1, ***Npepl1***	100	• Metabolism protein families<br>• Hydrolases<br>• Metallopeptidases	• NPEPL1 is predicted to have aminopeptidase and metalloexopeptidase activities that can be related to osteogenesis and matrix mineralization. Aminopeptidases have homogeneous activity patterns in various highly pure osteoblastic lines[3].<br>• Aminopeptidases in cells and matrix vesicles from fractures callus have ability to degrade natural proteoglycan substrates[4].<br>• Leucine aminopeptidase is highly active in young matrix-producing cells and appears to be involved in the degradation of matrix organic components to enable matrix mineralization[5].<br>• Glutamyl aminopeptidase is upregulated during osteogenic differentiation of rat bone marrow stromal cells, suggesting a role in osteogenesis[6].
P17125 - Transforming growth factor beta-3 proprotein, ***Tgfb3***	100	-	• Osteogenic stimulation of hASCs induces the expression of osteoblast-related genes, including *TGFB3*[7].<br>• TGFB3 controls the induction of bone formation by regulating the expression of *BMPs* via *Noggin* expression[8].<br>• Transcriptional mechanisms associated with guided bone regeneration include upregulation of growth and differentiation factors, like TGFB3. The *Tgfb*/*Bmp* and *Wnt* signaling pathways play important roles in the regenerative process[9].<br>• TGFB3 recruits MSCs to initiate bone formation via the TGFB signaling pathway and Smad3[10].<br>• A relationship between bone fragmentation and low *tgfb3* levels is evident in cave-dwelling fish[11].<br>• TGFB3 has been associated with human cranial malformations (oral clefts)[12].

E9Q557 – Desmoplakin, ***Dsp***	100*	• Cytoskeleton proteins<br>• Intermediate filament-binding proteins<br>• Exosomal proteins	• Desmoplakin is a protein involved in maintaining cytoskeletal architecture and cell stability, including in bone, that may behave as cell adhesion marker[13]. *DSP* also behaves as ECM remodeling gene[14].<br>• DSP is upregulated in the proteome of differentiating osteoblasts[15].<br>• Osteosarcomas display a decrease in genes involved with cytoskeleton stability, including *DSP*[14].
P35282 - Ras-related protein Rab-21, ***Rab21***	100*	• Membrane trafficking<br>• Exosomal proteins<br>• GTP-binding proteins<br>• Small (monomeric) G-proteins	• The Rab family of small GTPases regulates vesicular transport in all eukaryotes[16].<br>• Rab21 associates with the cytoplasmic domains of alpha-integrin chains, and its expression influences the endo/exocytic traffic of integrins. Rab21 knockdown impairs integrin-mediated cell adhesion and motility, whereas its overexpression stimulates cell migration. Rab21 activity regulates integrin-dependent adhesion to collagen[17].
Q6P5F7 - Protein tweety homolog 3, ***Ttyh3***	7.033	• Signalling and cellular processes protein families<br>• Transporters<br>• Pores ion channels	• The *tweety* gene encodes for Ca^{2+}-activated Cl^- channels[18,19]. Its human homologue (hTTYH3) is activated by elevated intracellular Ca^{2+} concentrations and predicted to have 6 transmembrane domains[19].<br>• Some patients with Turner Syndrome (characterized by low bone mineral status) have deletion of *TTYH3* gene[20].<br>• hTTYH3 channel showed complex gating kinetics and voltage-dependent inactivation[19] and hTTYH3 mRNA was found distributed in excitable tissues such as bone[21].<br>• Another member of the Tweety family, TTYH1, is likely downstream to Notch signalling[22] and osteoblasts require Notch signaling for proper differentiation[23].
F8WJ05 - Inter-alpha-trypsin inhibitor heavy chain H1, ***Itih1*** (unreviewed - TrEMBL)	5.246	-	• The ITI family encodes glycoproteins composed of three polypeptides: two heavy chains and one light chain. The light chain confers the protease-inhibitor function. The heavy chains are linked to hyaluronic acid, a covalent linkage that greatly improves ECM stability[24,25].<br>• ITI proteins are involved in the ECM dynamics, namely inducing matrix-stabilizing effects[26,27].
Q8C7E4 - Ribonuclease 4, ***Rnase4*** (unreviewed - TrEMBL)	3.239	• Hydrolases<br>• mRNA degradation factors	• Ribonuclease 4 is a highly conserved member of the RNAse superfamily and plays an important role in mRNA cleavage[28].<br>• (mi)RNAs have important functions in the epigenetic control of bone development and markedly regulate osteoblast differentiation, with osteogenic differentiation being blocked if Dicer (an endoribonuclease that regulates miRNA maturation) suffers knockdown[29].
P50446 - Keratin, type II cytoskeletal 6A, ***Krt6a***	2.918	• Cytoskeleton proteins<br>• Intermediate filaments<br>• Exosomal proteins	• Isoforms a and b of Keratin 6, a type II intermediate filament protein, regulate cell-matrix and cell-cell adhesion, and influence levels of other relevant proteins, including desmoplakin that is expressed at reduced levels in *Kr6a/Kr6b*-null cells[30].<br>• There is a structural relationship between an extracellular protein of mineralized tissue and keratin, with both sharing antigenic determinants[31].<br>• A flavonoid that promotes osteoblast mineralization *in vitro*, induces upregulation of cytokeratin[32].

hASCs: human adipose-derived mesenchymal stem cells; BMPs: bone morphogenetic proteins; MSCs: mesenchymal stem cells; ECM: extracellular matrix; GTP: guanosine-5'-triphosphate; ITI: inter-alpha-trypsin inhibitors.

These 8 upregulated proteins can be associated to different aspects of bone metabolism, such as:

- **Osteodifferentiation: TGFB3** is highly related to osteogenesis of stem and precursor cells[7,9,10] and **RNase4** may be involved in osteoblast differentiation via miRNAs regulation[29].
- **Matrix stability: DSP**, a protein involved in maintaining cytoskeletal architecture and cell stability[15]. **KRT6A** is an isoform of an intermediate filament that regulates cell-matrix and cell-cell adhesion[30]. **ITIH1** is a member of the ITI family of plasma protease inhibitors which contribute to ECM stability[25–27]. **Rab21**, a small GTPase regulating integrin-dependent cellular adhesion to collagen[17].

- **Matrix mineralization and voltage excitation**: **NPEPL1** is a predicted aminopeptidase and specific aminopeptidases are upregulated during osteogenic differentiation[6] and are suggested to be involved in the degradation of matrix organic components to enable mineralization[5]. The mRNA of **hTTYH3**, a Ca^{2+}-activated chloride channel, is distributed in excitable tissues, suggesting a possible relation with voltage excitation of osteoblasts[21]; its gene deletion was associated to a disease with a low bone mineralization phenotype[20].

Supplementary Table 2 | Microvesicles' proteins downregulated by daily high frequency stimulation. All proteins have high FDR confidence (<1%) and q-value = 0.0, and their levels considered significantly downregulated by the Volcano plot software; proteins concomitantly presenting adjusted p-value < 0.05 are shadowed (n=4).
AN, Uniprot accession number; GS, Gene Symbol; STIM / CTRL Abundance ratio, ratio of the relative protein abundance in *Stim* against *Ctrl* conditions. Functional hierarchies' classification retrieved from KEGG BRITE (genome.jp/kegg/brite.html). Possible roles determined through abstract mining in *PubMed* (pubmed.ncbi. nlm.nih.gov), using the terms 'bone', 'osteo', 'osseo', 'osteoblast', 'matrix' and 'mineralization'.

**AN - Protein name and GS**	**STIM / CTRL Abundance ratio**	**Functional hierarchies**	**Possible roles in bone metabolism or known involvement in bone cells' proliferation or differentiation**
P24549 - Retinal dehydrogenase 1, ***Aldh1a1***	0.01	• Oxidoreductases<br>• Retinol metabolism	• ALDH1A1 deficiency induces BMP2 and increases trabecular and cortical bone mass *in vivo*[33].<br>• ALDH1A1 participates in osteoclast differentiation via RANKL-ALDH1A1-NFATc1 axis[34] and in PPARγ-mediated bone loss[35].<br>• ALDH1A1 expression is transcriptionally inhibited by Smad4[36].
F6ZEW4 - Exportin-2, ***Cse1l*** (unreviewed - TrEMBL)	0.151	-	• Exportin-2 has not been studied regarding bone metabolism.<br>• Inhibition of exportin-5 significantly blocks osteoclast differentiation[37].<br>• Exportin-1 drives Runx2/Cbfa1 nuclear export upon supplementation of inorganic phosphate to MC3T3 cells[38].<br>• Exportin-1 blockage reduces proteases secretion, impairing the secretion of pro-osteolytic cytokines[39].<br>• Exportin-1 controls the nucleocytoplasmic trafficking of proteins and RNAs and its levels are significantly increased in osteosarcoma[40].
A2AHD1 - Cellular communication network factor 5, ***Ccn5*** (also known as ***Wisp2***) (unreviewed - TrEMBL)	0.157	• Signaling proteins	• WISP2/CCN5 is expressed in MSCs and its RNA expression declines during adipogenic differentiation[41].<br>• Secreted CCN5 (or WISP2) activates canonical WNT and maintains cells in an undifferentiated state[42,43].<br>• Intracellular WISP2 protein induces adipogenic commitment, through BMP4 induction (in a SMAD dependent manner) and subsequent PPARγ activation[44].<br>• CCN5/WISP2 is not required for normal bone formation[45].
P27046 - Alpha-mannosidase 2, ***Man2a1*** (also known as ***Mana2***)	0.158	• Glycanbiosynthesis and metabolism<br>• Glycosidases	• Although not specified for Man2a1/Mana2, alpha-mannosidase plasma levels are increased in patients with decreased bone mineralization[46].<br>• Higher enzymatic activity (including of alpha-mannosidase) occurs is chronic ear disease characterized by high bone resorption[47].
P51885 – Lumican, ***Lum***	0.165	• Proteoglycans<br>• ECM proteoglycans<br>• SLRP family class II	• LUM is a significant proteoglycan component of bone matrix, secreted by differentiating and mature osteoblasts[48,49].<br>• LUM is upregulated before the mineralization onset and then returns to baseline levels[50].<br>• LUM associates to fibrillar collagens and may regulate collagen fibrillogenesis[49].
Q9WVA4 – Transgelin-2, ***Tagln2***	0.175	• Membrane trafficking<br>• Actin-binding proteins	• TAGLN2 is an actin cross-linking protein[51] significantly downregulated in circulating monocytes (that can differentiate into osteoclasts) in females with postmenopausal osteoporosis and low bone mineral density[52].<br>• Bone marrow MSCs differentiate more rapidly into bone nodules but show a slower growth rate when expressing TAGLN2; significantly increased growth rates occur upon *TAGLN2* knockdown[53].<br>• TAGLN2 is highly downregulated in MSCs differentiated towards chondrogenic lineage[54].

G3UY13 - Interleukin-1 receptor accessory protein, ***Il1rap*** (unreviewed - TrEMBL)	0.175	-	• Soluble IL1RAP ameliorates collagen-induced arthritis potentially through inhibition of IL-1 signaling (an osteoclastogenesis regulator)[55].<br>• Administration of soluble IL1RAP also reduces bone erosion[56].<br>• Membrane-bound IL1RAP is downregulated when bone resorption occurs upon orthodontic tooth movement[57].<br>• *IL1RAP* is one of 9 top-hub genes that are deregulated in a pathology related to hyperosteoidosis, an excessive formation of osteoid[58].
Q9QUR6 - Prolyl endopeptidase, ***Prep***	0.177	• Metabolism protein families<br>• Hydrolases<br>• Serine endopeptidase	• PREP is reported to play a significant role on OA onset in joints since PREP activity is significantly higher in mice with spontaneous OA than in OA-free mice[59].<br>• Other endopeptidases, like the transmembrane zinc-endopeptidase MT1-MMP, MMP13 and PHEX, have roles in extracellular matrix remodeling, osteoblast differentiation and regulation of bone mineralization, respectively[60–62].
Q06806 - Tyrosine-protein kinase receptor Tie-1, ***Tie1***	0.178	• Protein kinases<br>•Transferring phosphorus-containing groups<br>• Receptor tyrosine kinases	• TIE1, a tyrosine-protein kinase receptor, is downregulated in conditions of NT3-induced *in vitro* osteogenesis. In this study, NT3 believed to promote skeletal remodeling, simultaneously enhanced osteogenesis markers such as OCN and Runx2[63].
K3W4L3 – Prosaposin, ***Psap*** (unreviewed - TrEMBL)	0.202	-	• PSAP is a precursor of saposin proteins, with no reported direct relationship to bone metabolism.<br>• Saposin-C (one potentially PSAP daughter protein) is specifically localized in arthritic paws and joints, increasing with disease progression[64].
P61082 - NEDD8-conjugating enzyme Ubc12, ***Ube2m***	0.203	• Ubiquitin mediated proteolysis<br>• Ubiquitin system<br>• Ubiquitin-conjugating enzymes	• UBE2M has not been studied regarding bone metabolism.<br>• One NEDD8-interacting protein, NUB1L, may contribute to p53 destabilization[65].<br>• Inhibition of the NEDD8 pathway prevents cell proliferation[66].<br>• Inhibitors of the NEDD8-activating enzyme induce DNA damage and cell cycle arrest[67].
O88200 - C-type lectin domain family 11 member A, ***Clec11a***	0.205	• Signalling and cellular processes protein families	• CLEC11A, also named Osteolectin, is secreted by osteoblast and promotes osteogenesis[68].<br>• CLEC11A activates *Wnt* pathway to maintain adult bone mass[69].<br>• Extracellular vesicles from umbilical cord MSCs were highly enriched in CLEC11A, a pro-osteogenic protein[70].<br>• CLEC11A promotes differentiation into mature osteoblasts *in vitro* and plays an important role in fracture repair[71].
Q2PZL6 - Protocadherin Fat 4, ***Fat4***	0.205	• Signal transduction<br>• Cell adhesion molecules<br>• Cadherin related	• Protocadherin FAT4 signals alongside DCHS1 to inhibit proliferation of mice early Runx2 osteogenic progenitors[72]. In human, mutations of the protocadherins FAT4 and DCHS1 result in Van Maldergem syndrome, characterized, in part, by craniofacial abnormalities[72].<br>• Another protocadherin, *PCDH7* is epigenetically regulated during osteoclasts formation; is induced by RANKL stimulation and activates osteoclastogenesis by promoting cell-cell fusion[73].
A0A1L1SSH9 – SPARC, ***Sparc*** (unreviewed - TrEMBL)	0.213	-	• SPARC (alias Osteonectin) is the most abundant non-collagenous protein of developing bone[74].<br>• This phosphorylated glycoprotein produced by osteoblasts is a component of the bone matrix[75]. SPARC binds Ca^{2+} and has affinity for hydroxyapatite and regulates cell shape, growth factor binding[76] and influences pathways involved in ECM assembly such as procollagen processing and collagen fibril formation[77].<br>• SPARC unique N-terminus contains the transglutaminase cross-linking site and the Ca^{2+} binding sites, suggesting a specific function in mineralization[78].<br>• SPARC was found in a wide variety of non-bone cell systems, in periods of rapid growth and proliferation[74].
Q9Z1R3 - Apolipoprotein M, ***Apom***	0.216	-	• Although there are few reports on ApoM, it is known to restrain lymphopoiesis when in a complex with the membrane sphingolipid Sphingosine-1-phosphate, by inhibiting the differentiation of bone marrow lymphocyte progenitors[79].<br>• In both humans and mice, circulating ApoE plays a strong inhibitory role in bone repair, reducing osteoblast differentiation, and reducing matrix mineralization and bone deposition[80].

P48759 - Pentraxin-related protein PTX3, ***Ptx3***	0.220	-	• PTX3 is a multifunctional glycoprotein involved in cell proliferation and migration, deposition and remodeling of the ECM in a variety of cells[81].<br>• PTX3 acts as a promoter of osteoblast differentiation and mineral matrix deposition[82].<br>• PTX3 is increasingly expressed during osteoblast maturation *in vitro*[83].<br>• PTX3 is highly expressed by precursor osteoblasts but not mature osteoblasts[84].
E9Q1Y3 - Apolipoprotein B-100, ***Apob*** (unreviewed - TrEMBL)	0.221	-	• ApoB lowers bone mineral density[85].<br>• The same inverse correlation occured under a randomized controlled clinical trial, where subjects with greatest decreases in ApoB experienced greatest increases in bone formation[86].
P12960 – Contactin-1, ***Cntn1***	0.229	• Cell adhesion molecules<br>• GPI-anchored proteins	• CNTN1 is involved in the regulation of TNFα for bone development[87].<br>• A contactin associated protein is located on the surface of osteogenic-committed cells[88].
E9PV24 - Fibrinogen alpha chain, ***Fga***	0.229	• Signalling and cellular processes protein families<br>• Exosomal proteins<br>• Glycosaminoglycan binding proteins<br>• ECM molecules	• FGA is widely known to be involved in hemostasis, as a component of the insoluble fibrin matrix following its cleavage by thrombin.<br>• FGA levels are increased over time in case of accelerated osteogenic orthodontics (tooth movement)[89].
P49182 - Heparin cofactor 2, ***Serpind1***	0.238	• Metabolism protein families<br>• Glycosaminoglycan binding proteins<br>• Exosomal proteins<br>• Signalling and cellular processes protein families	• SERPIND1 (aliases HCF2, HCII) is a proteolytic enzyme that degrades organic bone matrix, being important for osteoclastic bone resorption. *Serpind1* was identified among 5 novel RANKL-induced, NFATc1-dependent transcripts, involved in RANKL-induced osteoclast differentiation[90].
P53690 - Matrix metalloproteinase -14, ***Mmp14***	0.244	• Hydrolases<br>•Metalloendopeptidase	• MMP14 metalloendopeptidase is a collagenolytic enzyme located at the cell surface and implicated in ECM remodeling[91], that cleaves RANKL into active soluble RANKL[92] and confers pathological bone-resorbing capacity to macrophages[93].<br>• MMP14 contributes to OCN and ALP expression[94].<br>• In an osteocyte line, MMP14 is increased at day 3, before returning to baseline levels[95].<br>• Lumican (2nd protein in this table) inhibits the MSC differentiation through a decrease in MMP14 expression and activity[96].
Q9R0G6 - Cartilage oligomeric matrix protein, ***Comp***	0.246	• PI3K/Akt signalling pathway<br>• ECM-receptor interaction<br>• Focal adhesion<br>• Exosomal proteins<br>• Glycosaminoglycan binding proteins<br>• ECM molecules	• COMP is a large pentameric glycoprotein known to play a role in collagen secretion and fibrillogenesis[97].<br>• COMP molecular functions that may contribute to its role in skeletal disease, include: binding other ECM proteins, catalyzing the polymerization of type II collagen fibrils, and regulating chondrocyte proliferation[98].<br>• COMP is a non-collagenous ECM protein expressed in osteoblasts proximal to the growth plate[99].
Q9D0F9 - Phosphoglucomu tase-1, ***Pgm1***	0.247	• Pentose phosphate pathway<br>•Phosphotransferases	• PGM1 (alias CD68) is a marker for macrophage specificity. High levels of PGM1 expression in osteoclasts are associated with bone erosion[100].
Q6GT24 - Peroxiredoxin-6, ***Prdx6*** (unreviewed - TrEMBL)	0.269	• Oxidoreductases<br>• Hydrolases	• PRDX6 levels are decreased during osteogenic differentiation of hDPSC, and PRDX6 inhibits hDPSC cell growth and osteogenic differentiation, including ALP and mineralized nodule formation[101].<br>• PRDX6 may play an important part in later stages of osteogenic differentiation of rat's mandibular bone marrow stromal cells[102].

BMP: bone morphogenetic protein; RANKL: receptor activator of nuclear factor kappa-B ligand; NFATc: nuclear factor of activated T cells; PPAR: peroxisome proliferator-activated receptor; MSCs: mesenchymal stem cells; ECM: extracellular matrix; SLRP: small leucine-rich proteoglycan; OA: osteoarthrosis; MT1-MMP: membrane-type 1 matrix metalloproteinase; MMP13: matrix metallopeptidase 13; PHEX: phosphate-regulating neutral endopeptidase X-linked; NT3: neurotrophin-3; OCN: osteocalcin; NUB1L: NEDD8 ultimate buster-1 long; DCHS1: protocadherin-16; PCDH7: protocadherin-7; GPI: glycosyl phosphatidyl-inositol; TNFα: tumor necrosis factor alpha; ALP: alkaline phosphatase; PI3K/Akt: phosphatidylinositol 3-kinase / protein kinase B; hDPSC: human dental pulp stem cells.

Several of the 24 proteins **DReg** in secreted MVs upon HF stimulation (Supplementary Table 2) were clustered by their potential involvement in relevant bone metabolic functions:

- **Stemness or commitment into lineages other than osteogenic: CCN5** (or WISP2) maintains cellular undifferentiated states[42,43] or induces adipogenic commitment[44]. **TAGLN2** is downregulated in chondrogenic-differentiated MSCs[54]. **LUM** inhibits endothelial MSC differentiation through decreases in **MMP14** levels[96]. **PGM1** is a marker for macrophage specificity[100].
- **Osteogenic commitment and earlier osteodifferentiation states: CNTN1** regulates TNFα in bone development[87], and interacts with proteins on the surface of osteogenic-committed cells[88]. **Lum** is found in intermediate osteogenic states[48–50]. **CLEC11A** promotes osteogenesis[68], is highly enriched in extracellular vesicles from umbilical cord-derived MSC[70] and its deficiency in mice impairs osteogenic differentiation and delays fracture healing[68]. **UBE2M** and **CSE1L** are potentially involved in inhibiting osteoblast proliferation. **FGA** is increased with accelerated osteogenic orthodontics[89]. **PTX3** promotes osteoblast differentiation and is highly expressed by precursor but not mature osteoblasts[84]. **MMP14** contributes to OCN and ALP expression[94]. **FAT4** regulates the proliferation of early Runx2 osteogenic progenitors in mice[72]. **TIE1** is downregulated in conditions of neurotrophin-3-induced osteogenesis[63]. **PRDX6** inhibits growth and osteogenic differentiation of hDPSC, including their ALP activity and mineralized nodule formation[101].
- **Bone growth inhibitors and/or osteoclastogenesis promoters, leading to decreased bone mass: ALDH1A1** participates in PPARγ-mediated bone loss[35] and RANKL-induced osteoclastogenesis[34], and its expression is transcriptionally inhibited by Smad4[36], while its deficiency induces BMP2 and increases bone mass *in vivo*[33]. As **ALDH1A1**, **PGM1**, **SERPIND1** (aliases HCF2, HCII), PCDH7 (a protocadherin of the same family as **FAT4**), the collagenolytic metalloendopeptidase **MMP14**, are all involved in RANKL-mediated osteoclastogenesis and/or associated with bone erosion[34,73,90,92,100], as potentially are **TAGLN2**, **CSE1L**, **IL1RAP**, **PTX3** (Supplementary Table 2). **ApoM** is a potential bone growth inhibitor since other apolipoproteins, such as ApoB, ApoE, were reported to lower bone mineral density, inversely correlate with bone formation in humans, reduce osteoblast differentiation, strongly inhibit bone repair, and reduce matrix mineralization[80,85,86].
- **Extracellular matrix remodeling and bone mineralization: PREP** may be involved in ECM remodeling in osteoblasts, as reported for other endopeptidases[60–62]. **SPARC** (alias Osteonectin), the major non-collagenous component of the bone matrix, binds Ca^{2+}, has affinity for hydroxyapatite and influences pathways involved in ECM assembly[74,76,77]. **MAN2A1** is an alpha-mannosidase, and the circulating levels of alpha-mannosidases inversely correlate with bone mineralization in patients[46]. **COMP** and **LUM**, are two components of bone matrix that play a role in collagen secretion and fibrillogenesis[49,97]. **LUM** is upregulated before the mineralization onset and returns to baseline levels at the onset of mineralization[50]. During MSC differentiation, **LUM** was observed to influence the expression and activity of **MMP14**, a metalloendopeptidase collagenolytic cell surface protein (pro-osteoclastogenic)[96] also DReg by HF stimulation (Supplementary Table 2).

Supplementary References

1. Blair, H. C. *et al.* Osteoblast Differentiation and Bone Matrix Formation In Vivo and In Vitro. *Tissue Eng Part B Rev* **23**, 268–280 (2017).
2. Hasegawa, T. Ultrastructure and biological function of matrix vesicles in bone mineralization. *Histochem Cell Biol* **149**, 289–304 (2018).
3. Lucena, G. *et al.* Aminopeptidase activity profile in cultured human osteoblasts. *Biol Res Nurs* **15**, 56–61 (2013).
4. Einhorn, T. A. *et al.* Neutral protein-degrading enzymes in experimental fracture callus: a preliminary report. *J. Orthop. Res.* **7**, 792–805 (1989).
5. Nilsen, R. & Magnusson, B. C. Enzyme histochemical studies of induced heterotopic cartilage and bone formation in guinea pigs with special reference to acid phosphatase. *Scand J Dent Res* **89**, 491–498 (1981).
6. Wu, Y., Xiao, J., Wu, L., Tian, W. & Liu, L. Expression of glutamyl aminopeptidase by osteogenic induction in rat bone marrow stromal cells. *Cell Biol. Int.* **32**, 748–753 (2008).
7. Samsonraj, R. M. *et al.* Osteogenic Stimulation of Human Adipose-Derived Mesenchymal Stem Cells Using a Fungal Metabolite That Suppresses the Polycomb Group Protein EZH2. *Stem Cells Transl Med* **7**, 197–209 (2018).
8. Klar, R. M., Duarte, R., Dix-Peek, T. & Ripamonti, U. The induction of bone formation by the recombinant human transforming growth factor-β3. *Biomaterials* **35**, 2773–2788 (2014).
9. Ivanovski, S., Hamlet, S., Retzepi, M., Wall, I. & Donos, N. Transcriptional profiling of 'guided bone regeneration' in a critical-size calvarial defect. *Clin Oral Implants Res* **22**, 382–389 (2011).
10. Deng, M. *et al.* TGFβ3 recruits endogenous mesenchymal stem cells to initiate bone regeneration. *Stem Cell Res Ther* **8**, 258 (2017).
11. Gross, J. B., Stahl, B. A., Powers, A. K. & Carlson, B. M. Natural bone fragmentation in the blind cave-dwelling fish, Astyanax mexicanus: candidate gene identification through integrative comparative genomics. *Evol. Dev.* **18**, 7–18 (2016).
12. Vieira, A. R. *et al.* MSX1 and TGFB3 contribute to clefting in South America. *J. Dent. Res.* **82**, 289–292 (2003).
13. Yu, Y. *et al.* Short tail with skin lesion phenotype occurs in transgenic mice with keratin-14 promoter-directed expression of mutant CXCR2. *J. Leukoc. Biol.* **84**, 406–419 (2008).
14. Mintz, M. B. *et al.* An expression signature classifies chemotherapy-resistant pediatric osteosarcoma. *Cancer Res.* **65**, 1748–1754 (2005).
15. Simunovic, F. *et al.* Increased differentiation and production of extracellular matrix components of primary human osteoblasts after cocultivation with endothelial cells: A quantitative proteomics approach. *J. Cell. Biochem.* **120**, 396–404 (2019).

16. Tang, B. L. & Ng, E. L. Rabs and cancer cell motility. *Cell Motil. Cytoskeleton* **66**, 365–370 (2009).
17. Pellinen, T. *et al.* Small GTPase Rab21 regulates cell adhesion and controls endosomal traffic of beta1-integrins. *J. Cell Biol.* **173**, 767–780 (2006).
18. Halleran, A. D. *et al.* Characterization of tweety gene (ttyh1-3) expression in Xenopus laevis during embryonic development. *Gene Expr. Patterns* **17**, 38–44 (2015).
19. Suzuki, M. The Drosophila tweety family: molecular candidates for large-conductance Ca2+-activated Cl- channels. *Exp. Physiol.* **91**, 141–147 (2006).
20. Li, L. *et al.* Rare copy number variants in the genome of Chinese female children and adolescents with Turner syndrome. *Biosci. Rep.* **39**, (2019).
21. Suzuki, M. & Mizuno, A. A novel human Cl(-) channel family related to Drosophila flightless locus. *J. Biol. Chem.* **279**, 22461–22468 (2004).
22. Wu, H.-N. *et al.* Deficiency of Ttyh1 downstream to Notch signaling results in precocious differentiation of neural stem cells. *Biochem. Biophys. Res. Commun.* **514**, 842–847 (2019).
23. Regan, J. & Long, F. Notch signaling and bone remodeling. *Curr Osteoporos Rep* **11**, 126–129 (2013).
24. Bost, F., Diarra-Mehrpour, M. & Martin, J. P. Inter-alpha-trypsin inhibitor proteoglycan family--a group of proteins binding and stabilizing the extracellular matrix. *Eur. J. Biochem.* **252**, 339–346 (1998).
25. Hamm, A. *et al.* Frequent expression loss of Inter-alpha-trypsin inhibitor heavy chain (ITIH) genes in multiple human solid tumors: a systematic expression analysis. *BMC Cancer* **8**, 25 (2008).
26. Morcel, K. *et al.* Involvement of ITIH5, a candidate gene for congenital uterovaginal aplasia (Mayer-Rokitansky-Küster-Hauser syndrome), in female genital tract development. *Gene Expr.* **15**, 207–214 (2012).
27. Zhuo, L., Hascall, V. C. & Kimata, K. Inter-α-trypsin Inhibitor, a Covalent Protein-Glycosaminoglycan-Protein Complex. *J. Biol. Chem.* **279**, 38079–38082 (2004).
28. Hofsteenge, J., Vicentini, A. & Zelenko, O. Ribonuclease 4, an evolutionarily highly conserved member of the superfamily. *Cell. Mol. Life Sci.* **54**, 804–810 (1998).
29. Wu, H.-Y., Bi, R., Sun, T. & Xie, F. Deletion of Dicer blocks osteogenic differentiation via the inhibition of Wnt signalling. *Mol Med Rep* **19**, 2897–2905 (2019).
30. Wang, F., Chen, S., Liu, H. B., Parent, C. A. & Coulombe, P. A. Keratin 6 regulates collective keratinocyte migration by altering cell-cell and cell-matrix adhesion. *J. Cell Biol.* **217**, 4314–4330 (2018).
31. Lesot, H., Smith, A. J., Matthews, J. B. & Ruch, J. V. An extracellular matrix protein of dentine, enamel, and bone shares common antigenic determinants with keratins. *Calcif. Tissue Int.* **42**, 53–57 (1988).

32. Kumar, A. *et al.* Identification of kaempferol-regulated proteins in rat calvarial osteoblasts during mineralization by proteomics. *Proteomics* **10**, 1730–1739 (2010).
33. Nallamshetty, S. *et al.* Deficiency of retinaldehyde dehydrogenase 1 induces BMP2 and increases bone mass in vivo. *PLoS ONE* **8**, e71307 (2013).
34. Jia, Y. *et al.* Disulfiram suppressed ethanol promoted RANKL-induced osteoclastogenesis in vitro and ethanol-induced osteoporosis in vivo via ALDH1A1-NFATc1 axis. *Aging (Albany NY)* **11**, 8103–8119 (2019).
35. Nallamshetty, S. *et al.* Retinaldehyde dehydrogenase 1 deficiency inhibits PPARγ-mediated bone loss and marrow adiposity. *Bone* **67**, 281–291 (2014).
36. Hoshino, Y. *et al.* Smad4 Decreases the Population of Pancreatic Cancer-Initiating Cells through Transcriptional Repression of ALDH1A1. *Am. J. Pathol.* **185**, 1457–1470 (2015).
37. Tan, H. *et al.* Ursolic Acid Isolated from the Leaves of Loquat (Eriobotrya japonica) Inhibited Osteoclast Differentiation through Targeting Exportin 5. *J. Agric. Food Chem.* **67**, 3333–3340 (2019).
38. Fujita, T. *et al.* Phosphate provides an extracellular signal that drives nuclear export of Runx2/Cbfa1 in bone cells. *Biochem. Biophys. Res. Commun.* **280**, 348–352 (2001).
39. Gravina, G. L. *et al.* XPO1/CRM1-selective inhibitors of nuclear export (SINE) reduce tumor spreading and improve overall survival in preclinical models of prostate cancer (PCa). *J Hematol Oncol* **7**, 46 (2014).
40. Walker, C. J. *et al.* Preclinical and clinical efficacy of XPO1/CRM1 inhibition by the karyopherin inhibitor KPT-330 in Ph+ leukemias. *Blood* **122**, 3034–3044 (2013).
41. Schutze, N., Noth, U., Schneidereit, J., Hendrich, C. & Jakob, F. Differential expression of CCN-family members in primary human bone marrow-derived mesenchymal stem cells during osteogenic, chondrogenic and adipogenic differentiation. *Cell Commun. Signal* **3**, 5 (2005).
42. Robinson, J. A. *et al.* Wnt/beta-catenin signaling is a normal physiological response to mechanical loading in bone. *J. Biol. Chem.* **281**, 31720–31728 (2006).
43. Grünberg, J. R., Hammarstedt, A., Hedjazifar, S. & Smith, U. The Novel Secreted Adipokine WNT1-inducible Signaling Pathway Protein 2 (WISP2) Is a Mesenchymal Cell Activator of Canonical WNT. *J. Biol. Chem.* **289**, 6899–6907 (2014).
44. Hammarstedt, A. *et al.* WISP2 regulates preadipocyte commitment and PPARγ activation by BMP4. *Proc. Natl. Acad. Sci. U.S.A.* **110**, 2563–2568 (2013).
45. Jiang, J., Zhao, G. & Lyons, K. M. Characterization of bone morphology in CCN5/WISP5 knockout mice. *J Cell Commun Signal* **12**, 265–270 (2018).
46. Ceroni, J. R. M. *et al.* Clinical and radiological findings in Brazilian patients with mucolipidosis types II/III. *Skeletal Radiol.* **48**, 1201–1207 (2019).

47. Olszewska, E., Borzym-Kluczyk, M., Olszewski, S. & Zwierz, K. Catabolism of glycoconjugates in chronic otitis media with cholesteatoma. *J. Investig. Med.* **55**, 248–254 (2007).
48. Raouf, A. *et al.* Lumican is a major proteoglycan component of the bone matrix. *Matrix Biol.* **21**, 361–367 (2002).
49. Matheson, S., Larjava, H. & Häkkinen, L. Distinctive localization and function for lumican, fibromodulin and decorin to regulate collagen fibril organization in periodontal tissues. *J. Periodont. Res.* **40**, 312–324 (2005).
50. Takashi, M. *et al.* Differential gene expression of collagen-binding small leucine-rich proteoglycans and lysyl hydroxylases, during mineralization by MC3T3-E1 cells cultured on titanium implant material. *Eur. J. Oral Sci.* **113**, 225–231 (2005).
51. Han, M.-Z. *et al.* TAGLN2 is a candidate prognostic biomarker promoting tumorigenesis in human gliomas. *J. Exp. Clin. Cancer Res.* **36**, 155 (2017).
52. Zhang, L. *et al.* Network-based proteomic analysis for postmenopausal osteoporosis in Caucasian females. *Proteomics* **16**, 12–28 (2016).
53. Kuo, H.-C. *et al.* Use of proteomic differential displays to assess functional discrepancies and adjustments of human bone marrow- and Wharton jelly-derived mesenchymal stem cells. *J. Proteome Res.* **10**, 1305–1315 (2011).
54. Tay, L.-X., Lim, C.-K., Mansor, A. & Kamarul, T. Differential protein expression between chondrogenic differentiated MSCs, undifferentiated MSCs and adult chondrocytes derived from Oryctolagus cuniculus in vitro. *Int J Med Sci* **11**, 24–33 (2014).
55. Smeets, R. L. *et al.* Soluble interleukin-1 receptor accessory protein ameliorates collagen-induced arthritis by a different mode of action from that of interleukin-1 receptor antagonist. *Arthritis Rheum.* **52**, 2202–2211 (2005).
56. Smeets, R. L. *et al.* Effectiveness of the soluble form of the interleukin-1 receptor accessory protein as an inhibitor of interleukin-1 in collagen-induced arthritis. *Arthritis Rheum.* **48**, 2949–2958 (2003).
57. Chang, H.-H. *et al.* MMP-3 response to compressive forces in vitro and in vivo. *J. Dent. Res.* **87**, 692–696 (2008).
58. Ueyama, T. *et al.* Is gastrectomy-induced high turnover of bone with hyperosteoidosis and increase of mineralization a typical osteomalacia? *PLoS ONE* **8**, e65685 (2013).
59. Fukuoka, Y., Hagihara, M., Nagatsu, T. & Kaneda, T. The relationship between collagen metabolism and temporomandibular joint osteoarthrosis in mice. *J. Oral Maxillofac. Surg.* **51**, 288–291 (1993).
60. Xu, H. *et al.* Multiple essential MT1-MMP functions in tooth root formation, dentinogenesis, and tooth eruption. *Matrix Biol.* **52–54**, 266–283 (2016).

61. Li, L., Zhang, J. & Akimenko, M.-A. Inhibition of mmp13a during zebrafish fin regeneration disrupts fin growth, osteoblasts differentiation, and Laminin organization. *Dev. Dyn.* **249**, 187–198 (2020).
62. Liu, S., Tang, W., Zhou, J., Vierthaler, L. & Quarles, L. D. Distinct roles for intrinsic osteocyte abnormalities and systemic factors in regulation of FGF23 and bone mineralization in Hyp mice. *Am. J. Physiol. Endocrinol. Metab.* **293**, E1636-1644 (2007).
63. Zhang, J. *et al.* Neurotrophin-3 acts on the endothelial-mesenchymal transition of heterotopic ossification in rats. *J. Cell. Mol. Med.* **23**, 2595–2609 (2019).
64. Qi, X. *et al.* Saposin C coupled lipid nanovesicles specifically target arthritic mouse joints for optical imaging of disease severity. *PLoS ONE* **7**, e33966 (2012).
65. Bravo-Navas, S., Yáñez, L., Romón, Í. & Pipaón, C. Elevated FANCA expression determines a worse prognosis in chronic lymphocytic leukemia and interferes with p53 function. *FASEB J.* **33**, 10477–10489 (2019).
66. Flomerfelt, F. A. *et al.* Tbata modulates thymic stromal cell proliferation and thymus function. *J. Exp. Med.* **207**, 2521–2532 (2010).
67. Paiva, C., Godbersen, J. C., Berger, A., Brown, J. R. & Danilov, A. V. Targeting neddylation induces DNA damage and checkpoint activation and sensitizes chronic lymphocytic leukemia B cells to alkylating agents. *Cell Death Dis* **6**, e1807 (2015).
68. Yue, R., Shen, B. & Morrison, S. J. Clec11a/osteolectin is an osteogenic growth factor that promotes the maintenance of the adult skeleton. *Elife* **5**, (2016).
69. Shen, B. *et al.* Integrin alpha11 is an Osteolectin receptor and is required for the maintenance of adult skeletal bone mass. *Elife* **8**, (2019).
70. Hu, Y. *et al.* Human umbilical cord mesenchymal stromal cells-derived extracellular vesicles exert potent bone protective effects by CLEC11A-mediated regulation of bone metabolism. *Theranostics* **10**, 2293–2308 (2020).
71. Wang, M. *et al.* Molecular structure, expression, and functional role of Clec11a in skeletal biology and cancers. *J. Cell. Physiol.* (2020) doi:10.1002/jcp.29600.
72. Crespo-Enriquez, I. *et al.* Dchs1-Fat4 regulation of osteogenic differentiation in mouse. *Development* **146**, (2019).
73. Nakamura, H. *et al.* Global epigenomic analysis indicates protocadherin-7 activates osteoclastogenesis by promoting cell-cell fusion. *Biochem. Biophys. Res. Commun.* **455**, 305–311 (2014).
74. Termine, J. D. Cellular activity, matrix proteins, and aging bone. *Exp. Gerontol.* **25**, 217–221 (1990).
75. Termine, J. D. Non-collagen proteins in bone. *Ciba Found. Symp.* **136**, 178–202 (1988).
76. Robey, P. G. Vertebrate mineralized matrix proteins: structure and function. *Connect. Tissue Res.* **35**, 131–136 (1996).

77. Rosset, E. M. & Bradshaw, A. D. SPARC/osteonectin in mineralized tissue. *Matrix Biol.* **52–54**, 78–87 (2016).
78. Aeschlimann, D., Mosher, D. & Paulsson, M. Tissue transglutaminase and factor XIII in cartilage and bone remodeling. *Semin. Thromb. Hemost.* **22**, 437–443 (1996).
79. Blaho, V. A. *et al.* HDL-bound sphingosine-1-phosphate restrains lymphopoiesis and neuroinflammation. *Nature* **523**, 342–346 (2015).
80. Huang, R. *et al.* Lowering circulating apolipoprotein E levels improves aged bone fracture healing. *JCI Insight* **4**, (2019).
81. Tarantino, U., Feola, M., Celi, M. & Scimeca, M. PTX3: a new mediator of bone metabolism and osteoporosis. *Muscles Ligaments Tendons J* **7**, 200–201 (2017).
82. Parente, R. *et al.* The Long Pentraxin PTX3 in Bone Homeostasis and Pathology. *Front Immunol* **10**, 2628 (2019).
83. Grčević, D. *et al.* The Long Pentraxin 3 Plays a Role in Bone Turnover and Repair. *Front Immunol* **9**, 417 (2018).
84. Lee, E.-J. *et al.* PTX3 stimulates osteoclastogenesis by increasing osteoblast RANKL production. *J. Cell. Physiol.* **229**, 1744–1752 (2014).
85. Yerges-Armstrong, L. M. *et al.* Decreased bone mineral density in subjects carrying familial defective apolipoprotein B-100. *J. Clin. Endocrinol. Metab.* **98**, E1999-2005 (2013).
86. Bredella, M. A. *et al.* Effects of growth hormone administration for 6 months on bone turnover and bone marrow fat in obese premenopausal women. *Bone* **62**, 29–35 (2014).
87. Yang, C.-W. *et al.* An Integrative Transcriptomic Analysis for Identifying Novel Target Genes Corresponding to Severity Spectrum in Spinal Muscular Atrophy. *PLoS ONE* **11**, e0157426 (2016).
88. Li, C. *et al.* Neurexin Superfamily Cell Membrane Receptor Contactin-Associated Protein Like-4 (Cntnap4) Is Involved in Neural EGFL-Like 1 (Nell-1)-Responsive Osteogenesis. *J. Bone Miner. Res.* **33**, 1813–1825 (2018).
89. Wu, J. Q. *et al.* Magnetic Bead-based Salivary Peptidome Profiling for Accelerated Osteogenic Orthodontic Treatments. *Chin J Dent Res* **21**, 41–49 (2018).
90. Charles, J. F. *et al.* The collection of NFATc1-dependent transcripts in the osteoclast includes numerous genes non-essential to physiologic bone resorption. *Bone* **51**, 902–912 (2012).
91. Gutiérrez-Fernández, A. *et al.* Loss of MT1-MMP causes cell senescence and nuclear defects which can be reversed by retinoic acid. *EMBO J.* **34**, 1875–1888 (2015).
92. Cappariello, A. *et al.* Biotechnological approach for systemic delivery of membrane Receptor Activator of NF-κB Ligand (RANKL) active domain into the circulation. *Biomaterials* **46**, 58–69 (2015).
93. Kittaka, M. *et al.* Cherubism Mice Also Deficient in c-Fos Exhibit Inflammatory Bone Destruction Executed by Macrophages That Express MMP14 Despite the Absence of TRAP+ Osteoclasts. *J. Bone Miner. Res.* **33**, 167–181 (2018).

94. Barthelemi, S. *et al.* Mechanical forces-induced human osteoblasts differentiation involves MMP-2/MMP-13/MT1-MMP proteolytic cascade. *J. Cell. Biochem.* **113**, 760–772 (2012).
95. Prideaux, M. *et al.* MMP and TIMP temporal gene expression during osteocytogenesis. *Gene Expr. Patterns* **18**, 29–36 (2015).
96. Malinowski, M. *et al.* Effect of lumican on the migration of human mesenchymal stem cells and endothelial progenitor cells: involvement of matrix metalloproteinase-14. *PLoS ONE* **7**, e50709 (2012).
97. Posey, K. L., Coustry, F. & Hecht, J. T. Cartilage oligomeric matrix protein: COMPopathies and beyond. *Matrix Biol.* **71–72**, 161–173 (2018).
98. Posey, K. L. & Hecht, J. T. The role of cartilage oligomeric matrix protein (COMP) in skeletal disease. *Curr Drug Targets* **9**, 869–877 (2008).
99. Hofmann, A. *et al.* Extracorporeal shock wave-mediated changes in proliferation, differentiation, and gene expression of human osteoblasts. *J Trauma* **65**, 1402–1410 (2008).
100. Hansen, T. *et al.* Expression of cysteine proteinases cathepsins B and K and of cysteine proteinase inhibitor cystatin C in giant cell tumor of tendon sheath. *Mod. Pathol.* **14**, 318–324 (2001).
101. Park, K.-R. *et al.* Peroxiredoxin 6 Inhibits Osteogenic Differentiation and Bone Formation Through Human Dental Pulp Stem Cells and Induces Delayed Bone Development. *Antioxid. Redox Signal.* **30**, 1969–1982 (2019).
102. Wang, J., Li, Y., Wang, D., E, L. & Liu, H. [Effect of insulin on peroxiredoxin-6 in the osteogenic differentiation of rat's mandibular bone marrow stromal cells in high glucose]. *Shanghai Kou Qiang Yi Xue* **22**, 523–527 (2013).
