# Supplementary material for: Capacitive interdigitated system of high osteoinductive/conductive performance for personalized acting-sensing implants
Source: NPJ Regen Med. 2021 Nov 23;6:80. doi: 10.1038/s41536-021-00184-6 (PMC8611088; doi:10.1038/s41536-021-00184-6)
Supplement: Supplementary file 2 — Reporting Summary [file 41536_2021_184_MOESM2_ESM.pdf]

nature portfolio

Corresponding author(s): Marco P. Soares dos Santos and Sandra I. Vieira

Last updated by author(s): Sep 23, 2021

Reporting Summary

Nature Portfolio wishes to improve the reproducibility of the work that we publish. This form provides structure for consistency and transparency in reporting. For further information on Nature Portfolio policies, see our Editorial Policies and the Editorial Policy Checklist.

Statistics

For all statistical analyses, confirm that the following items are present in the figure legend, table legend, main text, or Methods section.

n/a	Confirmed	
☐	☒	The exact sample size (*n*) for each experimental group/condition, given as a discrete number and unit of measurement
☐	☒	A statement on whether measurements were taken from distinct samples or whether the same sample was measured repeatedly
☐	☒	The statistical test(s) used AND whether they are one- or two-sided<br>*Only common tests should be described solely by name; describe more complex techniques in the Methods section.*
☒	☐	A description of all covariates tested
☐	☒	A description of any assumptions or corrections, such as tests of normality and adjustment for multiple comparisons
☐	☒	A full description of the statistical parameters including central tendency (e.g. means) or other basic estimates (e.g. regression coefficient) AND variation (e.g. standard deviation) or associated estimates of uncertainty (e.g. confidence intervals)
☐	☒	For null hypothesis testing, the test statistic (e.g. *F*, *t*, *r*) with confidence intervals, effect sizes, degrees of freedom and *P* value noted<br>*Give P values as exact values whenever suitable.*
☒	☐	For Bayesian analysis, information on the choice of priors and Markov chain Monte Carlo settings
☒	☐	For hierarchical and complex designs, identification of the appropriate level for tests and full reporting of outcomes
☒	☐	Estimates of effect sizes (e.g. Cohen's *d*, Pearson's *r*), indicating how they were calculated

Our web collection on statistics for biologists contains articles on many of the points above.

Software and code

Policy information about availability of computer code

Data collection: n/a

Data analysis:
Immunoblot density analyses were performed with the Bio-Rad ImageLab software.
For the SEM-EDS analysis, calcium and phosphorous peaks were determined from the EDS spectra using the Esprit software.
Proteomic raw data were analyzed using the Proteome Discoverer 2.3.0.523 software (Thermo Scientific).
Gene Ontology analyses were performed using the FunRich software v3.1.3.
The hierachical clustering analysis was performed using the MetaboAnalyst 5.0 online-available platform.
Statistical analyses were performed using the GraphPad Prism 8.0.1 software.

For manuscripts utilizing custom algorithms or software that are central to the research but not yet described in published literature, software must be made available to editors and reviewers. We strongly encourage code deposition in a community repository (e.g. GitHub). See the Nature Portfolio guidelines for submitting code & software for further information.

Data

Policy information about availability of data

All manuscripts must include a data availability statement. This statement should provide the following information, where applicable:

- Accession codes, unique identifiers, or web links for publicly available datasets
- A description of any restrictions on data availability
- For clinical datasets or third party data, please ensure that the statement adheres to our policy

All relevant data will be made available by the authors upon request. The proteomic dataset generated during the study is available via the ProteomeXchange

Consortium with the dataset identifier PXD028551 and 10.6019/PXD028551. All datasets used for Gene Ontology enrichment analysis are detailed in the Methods section.

Field-specific reporting

Please select the one below that is the best fit for your research. If you are not sure, read the appropriate sections before making your selection.

☒ Life sciences ☐ Behavioural & social sciences ☐ Ecological, evolutionary & environmental sciences

For a reference copy of the document with all sections, see nature.com/documents/nr-reporting-summary-flat.pdf

Life sciences study design

All studies must disclose on these points even when the disclosure is negative.

Sample size	A minimum of 3 biological replicas were analyzed and most of the experiments (>90%) included at least 4 biological replicas.
Data exclusions	n/a
Replication	A minimum of 3 biological replicas were analyzed and most of the experiments (>90%) included at least 4 biological replicas. Technical replicas were used in all spectrophotometric evaluations.
Randomization	n/a
Blinding	n/a

Reporting for specific materials, systems and methods

We require information from authors about some types of materials, experimental systems and methods used in many studies. Here, indicate whether each material, system or method listed is relevant to your study. If you are not sure if a list item applies to your research, read the appropriate section before selecting a response.

Materials & experimental systems

n/a	Involved in the study
☐	☒ Antibodies
☐	☒ Eukaryotic cell lines
☒	☐ Palaeontology and archaeology
☒	☐ Animals and other organisms
☒	☐ Human research participants
☒	☐ Clinical data
☒	☐ Dual use research of concern

Methods

n/a	Involved in the study
☒	☐ ChIP-seq
☐	☒ Flow cytometry
☒	☐ MRI-based neuroimaging

Antibodies

Antibodies used	All antibodies used for the immunoblot and immunofluorescence analyses are described in the Methods section, including the supplier and the catalog number of each antibody and the dilutions used in each assay.
Validation	All antibodies are commercially available and were pre-validated by the suppliers and other authors (references in the suppliers' webpages).

Eukaryotic cell lines

Policy information about cell lines

Cell line source(s)	The sources for the immortalized cell line and the ASCs are detailed in the Methods section.
Authentication	Commercially available cell lines were validated by the suppliers and ASCs were validated by flow cytometry (validation protocol detailed in the Methods section, validation results presented in the Supplementary Figure 9).
Mycoplasma contamination	All cell lines are tested upon thawing for mycoplasma in our BSL-2 cell culture facility. All cell lines tested negative for mycoplasma contamination.
Commonly misidentified lines (See ICLAC register)	n/a

Flow Cytometry

Plots

Confirm that:

- [ ] The axis labels state the marker and fluorochrome used (e.g. CD4-FITC).
- [x] The axis scales are clearly visible. Include numbers along axes only for bottom left plot of group (a 'group' is an analysis of identical markers).
- [ ] All plots are contour plots with outliers or pseudocolor plots.
- [x] A numerical value for number of cells or percentage (with statistics) is provided.

Methodology

Sample preparation	Described in detail in the Methods section.
Instrument	Samples were analyzed in a BD Accuri C6 flow cytometer (BD Biosciences).
Software	Flow cytometry data were analyzed using the CellQuest v3.3 software (BD Biosciences).
Cell population abundance	The ASC populations presented an abundance >91% of cells positive for standard mesenchymal markers (characterization delailed in the Supplementary Figure 9).
Gating strategy	Gating strategies used were based on previous similar characterization analyses of ASCs performed by the group, as referred in the Methods section. Flow cytometry analysis of control unstained isolated cells (c.a. 33,000 events, with ~10,000 cells) using gating strategies for exclusion of artifacts like tissue debris, allowed to identify the relevant cellular population ('P2') based on size and complexity, by using the forward (FSC) and side (SSC) scatter dot plot. The boundaries between "positive" and "negative" cell populations were defined by analyzing the autofluorescence of control unstained ASCs in the FITC, PE, APC channels (fluorescence histograms).

- [x] Tick this box to confirm that a figure exemplifying the gating strategy is provided in the Supplementary Information.
